# Isolation of Four Microalgal Strains From the Lake Massaciuccoli: Screening of Common Pollutants Tolerance Pattern and Perspectives for Their Use in Biotechnological Applications

**DOI:** 10.3389/fpls.2020.607651

**Published:** 2020-12-09

**Authors:** Carolina Chiellini, Lorenzo Guglielminetti, Sabrina Sarrocco, Adriana Ciurli

**Affiliations:** ^1^Department of Agriculture, Food and Environment (DAFE), University of Pisa, Pisa, Italy; ^2^Italian National Research Council, Institute of Agricultural Biology and Biotechnology (IBBA), Pisa, Italy; ^3^Centre for Climate Impact, University of Pisa, Pisa, Italy

**Keywords:** freshwater microalgae, *Chlorella sorokiniana*, Ibuprofen, metals, herbicides, antibiotics

## Abstract

Aquatic ecosystems represent one of the largest reservoirs of phytoplankton accounting for most of the primary production of the Earth. The Lake Massaciuccoli located in Tuscany (Italy) is one of the largest swamps that in ancient times entirely covered the Versilia coastal plain. Despite its peculiar features, especially the eutrophic characteristics, its native microalgal consortia have never been explored up to now. In this work, we isolated and described four autochthonous microalgal strains from different sites in the lake (FB, Idr, CL_Sc, and CL_Ch); the four microalgal strains were identified within the *Chlorella sorokiniana* clade. We exposed them to ten of the most common or emerging environmental contaminants in order to describe their preliminary response to the tested substances: five metals (As, Fe, Ni, Cu, and Zn), two herbicides (Metolachlor and Sethoxydim), two antibiotics (Ciprofloxacin and Benzylpenicillin) and a non-steroidal anti-inflammatory drug (Ibuprofen). Physiological response of the strains highlighted intraspecific differences; strain CL_Sc was the most tolerant in presence of metals while strain Idr was the most sensitive. All strains were sensitive to sethoxydim and tolerant to metolachlor at all the tested concentrations. Strains FB and Idr were the most sensitive in presence of Ibuprofen while strain CL_Ch was the most sensitive to the highest Benzylpenicillin concentration. Resistance pattern of strain Idr somehow reflects both the phylogenetic and the geographic “isolation” from all other three strains. Finally, optical microscope observation confirmed some differences also in the microalgae morphological aspect. Overall, all the strains showed interesting responses in presence of high concentrations of the tested substances, representing putative interesting candidates for water remediation in wastewater treatment plants.

## Introduction

Aquatic ecosystems represent one of the largest reservoirs of biodiversity, mainly phytoplankton. Phytoplankton accounts for most of the primary production of our planet and it is at the base of the entire aquatic food chain (Muller-Feuga, 2013). Freshwater environments (i.e., rivers, lakes, and shallow waters) hosts the largest microalgal biodiversity in terms of species, size and shapes (Sheath and Wehr, 2015). This almost unlimited resource of microalgal biodiversity represents a promising advantage for humanity under many fields of action. To this purpose, in the last decades, new perspectives for the use of microalgae in different areas of application have been opened, especially related to pollution matters, environmental challenges, food production. Accordingly, the use of microalgae in the production of new renewable energy sources – biofuels – is largely studied (i.e., Ahmad et al., 2011), as well as the potential use of microalgal biomass for food production (Costa and de Morais, 2013), chemicals production (Cohen, 1999), and also as bioindicators of environmental status (Amengual-Morro et al., 2012). Interestingly, one of the major application of microalgae is the remediation of contaminated environmental matrices (phycoremediation) (e.g., Pacheco et al., 2020). Microalgae are considered an important resource in wastewater treatment plant (Gonçalves et al., 2017), not only for municipal wastewater (Boelee et al., 2011), but also for industrial ones (Wang et al., 2016). Phycoremediation process may occur both using living biomass or death biomass. In the first case, living organisms need a minimum amount of nutrients (that can be recovered directly from the wastewater without any external addition); on the other side, dead biomass is sometimes recommended because it does not require nutrients nor oxygen (Kumar et al., 2015), and it reduces the toxic effects that the pollutants may cause to the organisms (Oliveira et al., 2011). In living algal cells, the ability to treat wastewater depend on the growth rate, which directly determines the biomass concentration, and influences the total biosorption capacity of contaminants. Microalgae can be applied for the removal of the most common environmental pollutants as metals (Leong and Chang, 2020) and hydrocarbons (Luo et al., 2014), but also for newly emergent contaminants such as common pharmaceuticals (Xiong et al., 2018) and herbicides (González-Barreiro et al., 2006). Metal contamination in the environment represents one of the most serious threats to human health; phycoremediation of metals takes place through two main uptake systems: the first one, called biosorption, is defined as the binding of metallic species to the cell surfaces; the second, called bioaccumulation, is an active intracellular uptake across the cell membrane, that requires living biomass (i.e., Tam and Wong, 1997). Biosorption can occur both as an active process or as a passive process, even if according to many authors (i.e., Kumar et al., 2015; Yadav et al., 2017), it is a method of uptake that is independent of the biological metabolic cycle (passive uptake). Microalgal biosorption with death and living biomass is also one of the mechanisms used for the removal of Ibuprofen from wastewater (Santaeufemia et al., 2018). Despite most of the deleterious effects of the diffusion of metal and herbicide contamination in the aquatic ecosystem are well known and documented, the effect of the newly emerging contaminants (drugs, pharmaceuticals, antibiotics, personal care products) is still under study. The presence of metals in aquatic ecosystems can come from both natural and anthropogenic sources. The main sources of metal pollution are the fossil fuels combustion, mining and smelting of metalliferous industry activities, municipal wastes, sewage, the use of pesticides and fertilizers (Rai, 2008). Metal environmental contamination can persist over time and can be accumulated in soils, water bodies or in the air, disrupting physiological functions in several biological systems (Ali et al., 2013). Metal pollution can cause several diseases also for humans, leading to the insurgence of neurobehavioral disorders, but also cancers, kidney damage, autoimmunity, and even death (Duruibe et al., 2007). Also, the extended use of herbicides for agronomic purposes and their continuous dischargement into aquatic environments through the surface runoff (Matamoros and Rodríguez, 2016), can have several negative consequences for water ecosystems. Since herbicides are specifically designed to kill unwanted plants, the most sensitive groups of aquatic non-target organisms are aquatic plants and algae. Plants might be affected from the presence of herbicides even at very low dosage, showing damaged growth and reproduction (Boutin et al., 2000). Herbicides have been also found to affect species composition and community structure of benthic algal assemblages in natural aquatic ecosystems (Sabater et al., 2007), and consequently, since microalgae are at the base of most of food-webs in the aquatic environment, to have negative effect on the functioning of aquatic ecosystems. Fortunately, recent studies highlighted the ability of freshwater microalgal strains to tolerate and decontaminate herbicides from the environments (González-Barreiro et al., 2006), and so opening new perspectives for their removal using biological strategies (Chekroun et al., 2014). Finally, also the presence of emerging contaminants such as pharmaceuticals and personal care products, is persistent in the environment since wastewater treatment plants are not designed to remove them (Ternes et al., 2004). The effect of emerging contaminants on the ecosystems is not yet fully understood, hence, the need to find new methods for their removal from wastewater is increasing.

The Lake Massaciuccoli is located in the center of Italy, Tuscany. With a surface area of 6.9 km^2^, it represents one of the largest swamps that in ancient times entirely covered the Versilia coastal plain. From an environmental point of view, the Lake Massaciuccoli has been largely studied in the past decades for its eutrophication problems (i.e., Ciurli et al., 2009), for mapping the biodiversity of plant species (Lastrucci et al., 2017; Bertacchi et al., 2019), to study fish parasites (Macchioni et al., 2015) and also to investigate on the ciliate (protists) community harboring the lake (Mori et al., 1998). Despite its peculiar features, especially the eutrophic characteristics, the microalgal consortia of the Lake Massaciuccoli have never been explored up to now. In this work, we aimed at isolating autochthonous microalgal strains from different sampling sites of the lake, starting for the first time a study on the phytoplanktonic community of this environment. Once isolated, native strains were described by means of optical microscopy and phylogenetic analysis, and also for their tolerance patterns after the exposure to the most common and emerging environmental contaminants: metals, herbicides (Metolachlor and Sethoxydim) and pharmaceutical products (Ibuprofen, Ciprofloxacin, and Benzylpenicillin). The four microalgal strains have been identified through molecular techniques as belonging to the *Chlorella* sp. genus, closely related to *Chlorella sorokiniana* species. The study allowed the individuation of those *Chlorella* sp. strains showing at laboratory-scale the best tolerance patterns against these common environmental contaminants.

## Materials and Methods

### Study Area

The Lake Massaciuccoli is mainly located in the municipality of Massarosa and Viareggio (Lucca Province), and partly in the municipality of Vecchiano (Pisa Province); it is included in the Regional Park Migliarino-San Rossore-Massaciuccoli, and its surface area covers 1908.01 ha. Waters depth rarely exceed 2.5 m, reaching in almost all the lake depths between 1 to 2.5 m (Spandre and Meriggi, 1997). The area is considered one of the most important Tuscan Ramsar wetlands and has been included within the Natura 2000 network as Special Area of Conservation (SAC) named “Lago e Padule di Massaciuccoli,” code IT5120017 (Lastrucci et al., 2017). The whole area is characterized by a dense network of channels surrounding the lake; the most important tributaries are the “Barra” and “Barretta” channels, “Fosso Confine,” “Fosso La Vite,” and “Fossa Nuova.” There is just one emissary, the Burlamacca channel (Lastrucci et al., 2017). The area surrounding the lake is reclaimed by five idrovoras, which are draining machines aimed at removing the runoff water from the land. In this case, the removed water is flowed in the Barra channel. Lake and channels hydraulic regimes are strongly influenced by environmental conditions such as seasonal rainfall, temperature and by the contribution of the aquifer (Spandre and Meriggi, 1997). Historically, it is well known that the main problem of Lake Massaciuccoli is eutrophication (i.e., Ciurli et al., 2009) that is the abundance of nutrients in the aquatic environment, mainly nitrates and phosphates. The main cause of the eutrophication problem is human activity of the surrounding areas mainly started during the 1950–1960s, and more in details, the sewage waters derived from residential buildings and from productive processes, the peat mineralization and the use of agricultural land. One of the direct consequences of eutrophication is the abnormal increase in phytoplankton, the impoverishment of the submerged macrophytic community followed by a decrease of gas exchange and water clearness (Lastrucci et al., 2017).

### Isolation of Microalgal Strains, Growth Conditions

**FIGURE 1:**
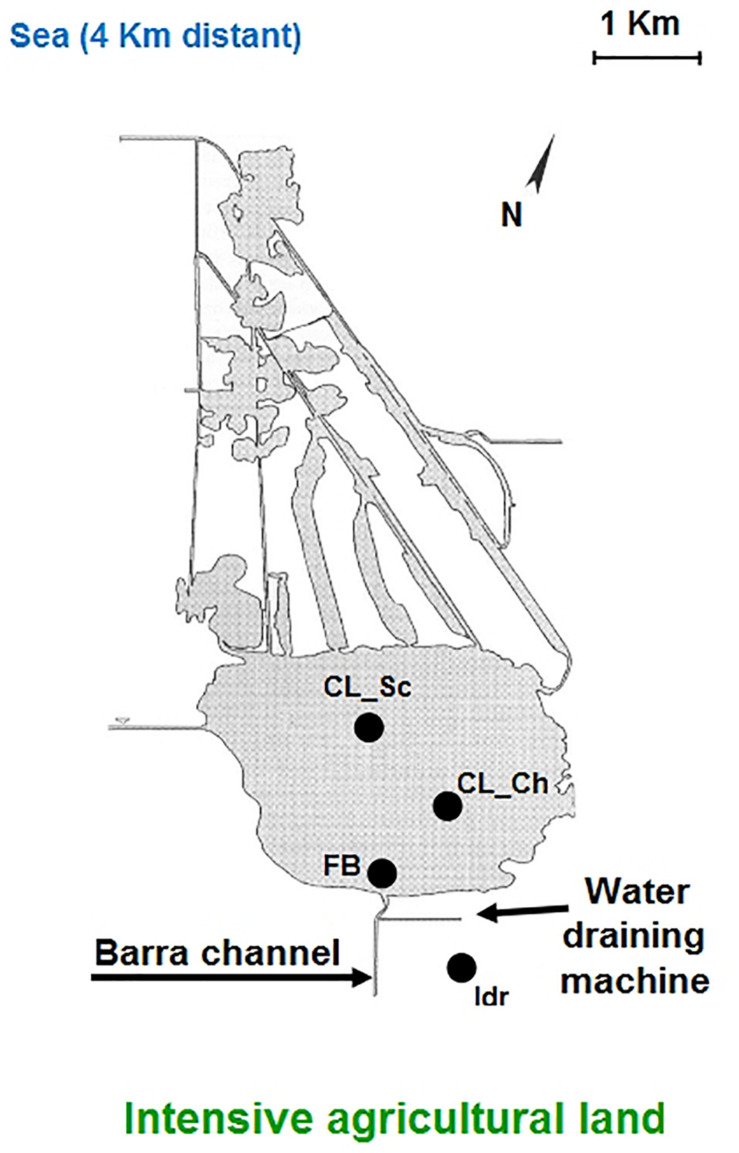
Schematic representation of the Massaciuccoli basin structure and geographic position; the four sampling sites are indicated in the figure.

Four different microalgal strains were isolated from water samples collected in the Lake Massaciuccoli (Tuscany, Italy), in four different sites within the whole area, during Spring 2019 (Figure 1). Water samples were collected in 1 liter sterile plastic bottles, at 20–30 cm depth and immediately brought to the laboratory for processing. Two strains, namely “CL_Sc” and “CL_Ch” were isolated from two distinct samples collected in the center of the lake; the sample from which “Idr” strain was isolated, was collected in proximity of the idrovora, where dewatering pumps collects the agricultural run-off. Finally, strain “FB” was isolated from a sample collected in proximity of the mouth of the Barra channel, collecting all the runoff water. Isolation of the strains has been carried out after an enrichment of the original sample; the culture enrichment was carried out by diluting the original water samples (1/1 v/v) in Tris-Ammonium Phosphate (TAP) medium (Gorman and Levine, 1965) and maintaining them for 2 weeks in the growth chamber under controlled temperature (24/22°C), and under a 16/08 h day-night cycle with PPFD of 120 μmol photons m^–1^ s^–1^ from cool-white light lamps (Gavita Lep 330 Plasma fixtures, Gavita Holland Light Emitting Plasma, Netherlands). After 2 weeks, each sample was streaked on a TAP agar plate (100 μl sample) in triplicate; plates were then kept in the growth chamber in the same conditions described above; this process was further repeated until the colonies appeared well isolated and purified (i.e., a single visible morphology indicating the presence of a single strain). Four colonies were randomly chosen from the plates derived from different water samples and pre-inoculated in liquid TAP medium (100 ml). Four massive dense pre-cultures (500 ml) were obtained from each pre-inoculum and used for the experiments described in this research. As previously reported (Chiellini et al., 2020), a periodic maintenance of the microalgal culture was performed by means of the substitution of the exhausted medium with the fresh one almost every 2 weeks. Periodical observation under optical microscopy (Nikon TMS-F 301434, Japan) were carried out in order to monitor the general health status of the culture and to check the absence of possible eukaryotic contamination. The culture set up and maintenance was performed under sterile condition for the whole experimental duration (i.e., sterile media, sterile glass-tubes and sterile flasks and bottles, use of the laminar flow).

### Light Microscope *in vivo* Observation

Aliquots (20 μl) of each microalgal culture, obtained as described before, were submitted to microscopic observations in order to visually assess phenotypical differences, as well as to take measures of each strain. Microscopic observations were performed by using a Dialux 22 light microscope (Leitz, Stoccarda, Germany) in immersion - under mineral oil – at 100× magnification. The microscope was equipped with a Leica DC450 C (Leica, Wetzlar, Germany) digital camera. Images were handled with the software Leica LASX (Leica Application Suite X).

### Identification of Microalgal Strains and Phylogenetic Analysis

The four microalgal strains were molecularly identified through the amplification and sequencing of the 18S rRNA gene, as described in Chiellini et al. (2012). Briefly, total genomic DNA was obtained from the four strains according to the protocol described by Saba et al. (2016), from the frozen cellular pellets. Pellets were obtained by centrifuging 2 ml of each mono-strain culture (5′ at 14,000 *g*) derived from a single colony grown on solid TAP medium. Amplification of the 18S rRNA gene was performed using primers 18S F9 5′-CTG GTT GAT CCT GCC AG-3′ (Medlin et al., 1988) and reverse 18S R1513 Hypo 5′-TGA TCC TTC YGC AGG TTC-3′ (Petroni et al., 2002). A 25-μl PCR reaction was set up using 0.25 mM deoxynucleoside triphosphates (2.5 mM each), 0.6 pmol μl^–1^ of each primer, 1 μl of template DNA, and 0.03 u μl^–1^ of taq polymerase (Promega, United States). Thermocycling was performed using a Bio-Rad Gene Cycler Thermal Cycler. Amplicons were purified by ethanol/EDTA/Na-acetate precipitation as follows: to 20 μl amplicon, add 2.25 μl EDTA (125 mM), 2.25 μl Na-acetate (3M, pH 5.6) and 50 μl cold ethanol (95%); leave 15 min at room temperature, and centrifuge 15 min at maximum speed; remove the supernatant without moving the pellet; wash with 100 μl cold ethanol (70%) and leave 10 min at room temperature; centrifuge 10 min at maximum speed and remove the supernatant; finally, air-dry and resuspend in 20 μl sterile distilled water.

The purified amplicons were sequenced using the same primers as for the amplification (i.e., F9 and R1513 Hypo) from the company BMR Genomics (Padova, Italy); and the obtained sequences were deposited in GeneBank.

The obtained sequences were separately submitted to NCBI Blast analysis (Altschul et al., 1997) to determine their preliminary affiliation through comparison with all the sequences present in the international databases. After an accurate review of the BLAST results, a selection of 43 high quality sequences was performed for the amplified region and the phylogenetic analysis were conducted as described in Chiellini et al. (2018). Briefly, the BioEdit Software (Hall, 1999) was used to align the selection of sequences together with the sequences obtained in this work, for a total of 47 sequences. MEGA5 Software (Tamura et al., 2011) was used for phylogenetic tree construction by means of the Maximum Likelihood and the Neighbor Joining algorithms. For Maximum Likelihood method, the robustness of the inferred trees was evaluated by 500 bootstrap resampling and the parameters chosen for the analysis were the following: Model/Method = General Time Reversible model; Rates among sites = Gamma distributed with invariant sites (G + I); Gaps = Use all sites; ML heuristic method = Nearest neighbor Interchange (NNI); Branch swap filter = Strong. The Neighbor Joining algorithm was applied following Chiellini et al. (2019), using Tamura-Nei Model, partial-deletion, Nearest-Neighbor Interchange Heuristic Method, and 1000 bootstrap resampling to evaluate the robustness of the inferred trees.

### Metal Tolerance

The tolerance toward a panel of five different metals was tested as described in Chiellini et al. (2020); briefly, the microalgae were collected from the original microalgal cultures (5 ml volume) and then centrifuged (1,000 *g* at room temperature for 10 min, Speedmaster 14R, Euro Clone, Milano, Italy), rinsed with sterile distilled water and resuspended in fresh TAP medium, in a total volume of 1 l. Three different concentration levels were tested for each metal (hereinafter renamed as “Low,” “Medium,” and “High,” in order to distinguish among the three concentrations tested). Each concentration was tested in triplicates, and three negative controls (the microalgal culture without any metal solution) were established in every experiment, resulting in 48 tests for each microalgal strain (5 metals × 3 concentrations × 3 replicates, + 3 control). Every test was conducted on a total volume of 20 ml in sterile glass tubes (50 ml volume), under the same growth conditions in which microalgae cultures were kept during the previous months/years. The metal solution was directly added to the microalgae culture (in sterile conditions) without macroscopic change in the pH values. Table 1 resumes the chosen metals and the applied concentrations. As previously reported (Chiellini et al., 2020), metal concentrations were chosen by comparing metal concentration measured in some of the most contaminated sites in the world (e.g., Schwartzbach and Shigeoka, 2017).

**TABLE 1:** List of metal used in the experiment, from Chiellini et al. (2020).

Metal	Salt	pH of the stock solution	Concentration stock solution (mM)	Low final concentration	Medium final concentration	High final concentration
Cu	CuCl_2_ × 2H_2_O	5.07	500	2.5 mM	5 mM	10 mM
Zn	ZnSO_4_ × 7H_2_O	5.6	500	5 mM	10 mM	15 mM
As (III)	NaAsO_2_	9.98	100	0.5 mM	1 mM	2.5 mM
Fe	Fe(SO_4_) × 7H_2_O	2.84	500	2.5 mM	5 mM	10 mM
Ni	NiSO_4_ × 6H_2_O	5.95	500	2.5 mM	5 mM	10 mM

*Three different concentrations have been applied for each metal and are listed in the table as “Low,” “Medium,” and “High” concentration. The pH of each stock solution has been indicated as well.*

After 1 week exposure, the following growth parameters were evaluated in order to assess the strain tolerance in presence of metals: chlorophyll a, chlorophyll b, total chlorophyll and carotenoids. For metal tolerance tests, row data from each replicate was used to calculate the average value for each pigment in each test; then, this average value was expressed as % compared to the pigment content of C. This last, was considered to represent the 100%, according to Chiellini et al. (2020). Greater chlorophyll content corresponded to a greater microalgal growth, while an increment in carotenoid content coupled with a decrease in chlorophyll content was interpreted as an index of stress and sufferance, compared to the optimal growth condition of the C. According to Chiellini et al. (2020), the Optical Density (O.D.) measured a 750 nm was used as a growth parameter in the analysis of metal tolerance only in the case of arsenic and nickel, since all the other metal solutions were colored (iron, copper) or turbid (zinc) due to the presence of the metal solution. Results were plotted using the “ggplot2” and “ggpubr” packages of the R software (Wickham, 2009), for boxplot and histogram construction and for statistical tests respectively (student *t*-test).

### Tolerance of Antibiotics, Herbicides and Ibuprofen and Growth Parameter Measures

#### Antibiotics

To test whether the presence of antibiotics might affect the growth of microalgae, strains were streaked on TAP agar plates containing three different concentrations of Ciprofloxacin and Benzylpenicillin (Penicillin G, 0.1, 1, and 10 μg/ml); tests were performed in triplicate. The growth of the strains on antibiotics was compared with a control plate, in which no antibiotic was added in the medium, according to Mengoni et al. (2014).

#### Herbicides

The two tested herbicides were Metolachlor and Sethoxydim. Metolachlor was tested at 100, 200, and 300 μg l^–1^, according to the toxicity level in microalgae listed in King et al. (2017). Sethoxydim was tested at concentrations 0.05, 0.08, and 0.1% v/v, following the study of Smythers et al. (2019). Each herbicide tolerance test was conducted for 1 week, on a total volume of 10 ml in sterile glass tubes (30 ml capacity), under the same growth conditions in which microalgae cultures were kept during the previous months/years. The herbicide solution was directly added to the microalgae culture (in sterile conditions) reaching the requested final concentrations. Each concentration was tested in triplicate, and three control tests (algal culture without any herbicide addiction) were included in the analysis. At the end of the experiment, microalgal cultures were sampled to measure the following growth physiological parameters, described in detail in the next paragraph: dry weight, total chlorophyll content, total carotenoids content and O.D.

#### Ibuprofen

In this work, the microalgal strains were exposed to high (0.20 and 1 mg l^–1^) and environmentally relevant (0.02 mg l^–1^) concentrations of Ibuprofen, according to Di Baccio et al. (2017). The Ibuprofen test was conducted in the same conditions as the herbicides test (see previous paragraph). At the end of the weekly exposure, microalgal cultures were sampled to measure dry weight, total chlorophyll content, total carotenoids content and O.D.

#### Growth Parameters and Photosynthetic Pigment Content Measures

The culture Optical Density (O.D.) were measured at 750 nm, that is the wavelength value outside the range of absorbance of the pigments (Griffiths et al., 2011) or not interfering with them, by using a UV-1800 Spectrophotometer (Shimadzu, Japan). The dried weight (biomass) was measured by an analytical balance (Ohaus^®^ Pioneer^TM^ Plus Model PA114C) in the microalgal cultures (both tests and control) after 48 h air-dry at room temperature, starting from 2 ml sample test; the biomass was expressed as g l^–1^.

The measured photosynthetic pigments (chlorophyll a -Chl-*a*-, chlorophyll b -Chl-*b*-, total chlorophyll -Chl-Tot- and carotenoids) were extracted in 100% methanol (Sigma Aldrich, MI, United States), according to Chiellini et al. (2020). Briefly, 1 ml of each sample-test was centrifuged (1500 rpm, 5 min at 4°C) and the pellet was resuspended in 1 ml methanol (Sigma Aldrich, MI, United States) and submitted to 10 min sonication (Branson 1210, Bransonic Ultrasonic Cleaner, United States). After this treatment, samples were kept in the dark at 4°C overnight, and subsequently centrifuged (12,000 rpm, 5 min). All the centrifuges were performed in a Speedmaster 14R, Euro Clone, Milano (Italy). Finally, the absorbance of the supernatant was spectrophotometrically analyzed (UV-1800 Spectrophotometer, Shimadzu, Japan) with regards to the blank at 665.2, 652.4, and 470.0 nm, according to the equations indicated in Lichtenthaler (1987).

All the results were analyzed and plotted using the “ggplot2” and “ggpubr” packages of the R software (Wickham, 2009), for boxplot construction and for statistical tests respectively (student *t*-test).

## Results

### Light Microscope *in vivo* Observation

**FIGURE 2:**
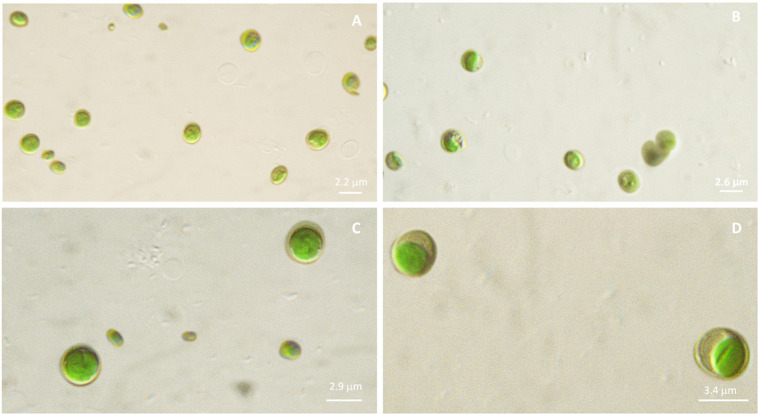
Microscopic observation (100× magnification, under mineral oil) of microalgal cultures in liquid TAP medium. **(A)**: Idr; **(B)**: FB; **(C)**: CL_Ch; **(D)**: CL_Sc.

Microscopic observations performed on microalgal cultures (liquid TAP medium) allow to visualize phenotypic differences among the four strains. In details, diameter of each sample resulted different, with increasing values, on average, from Idr (2.2 μm), FB (2.6 μm), CL_Ch (2.9 μm) to CL_Sc (3.4 μm), as shown in Figure 2. In addition, also morphology seems to be quite different, with CL_Ch and CL_Sc strains showing a more intense green that the other two.

### Molecular Identification of Microalgal Strains

The amplification and sequencing of the 18S rRNA gene of the four microalgal strains, allowed to obtain three partial sequences of 695, 810, and 833 bp for strains CL_Ch, CL_Sc and FB respectively, and an almost complete sequence of 1694 bp for strain Idr. Sequences were deposited with the following accession numbers: MT992788 (CL_Ch), MT992789 (CL_Sc), MT992790 (FB), and MT992791 (Idr). Each sequence was submitted to NCBI Blast analysis and the results are shown in Supplementary Tables 1A–D. As it is shown in all the tables, BLAST results highlight a similarity of all the four microalgal strains with *Chlorella sorokiniana*. Within the first 10 queries provided for each of our strain, *C. sorokiniana* NKH18 (Acc.Nr. LC505550), and *Micractinium* sp. ACSSI (Acc. Nr. MK235183) are the ones sharing similarities with all our isolates, with different identity percentages. Sequence obtained from strain “FB” shows the highest percentage of similarity (98.45%) in presence of 100% query coverage. On the other side, strain CL_Ch show the lowest level of similarity (95.03%) in presence of the lowest query coverage (95%).

### Phylogenetic Analysis

**FIGURE 3:**
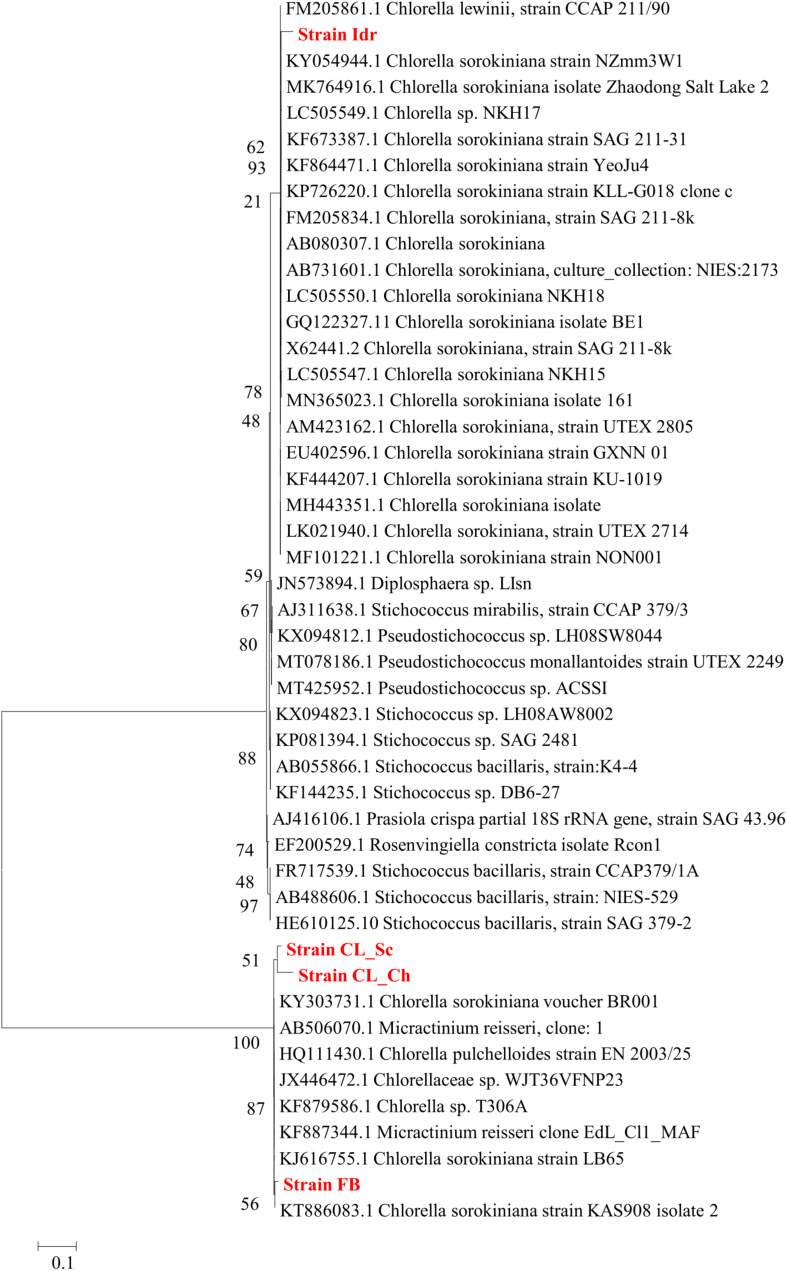
Phylogenetic tree reconstruction obtained with Maximum Likelihood method on a total of 43 high quality sequences selected from the most similar to the sequences obtained for the four strains analyzed in this work (Acc. N. MT992788-MT992791). Reconstruction was performed with 500 bootstrap resampling and the following parameters: Model/Method = General Time Reversible model; Rates among sites = Gamma distributed with invariant sites (G + I); Gaps = Use all sites; ML heuristic method = Nearest neighbor Interchange (NNI); Branch swap filter = Strong.

**FIGURE 4:**
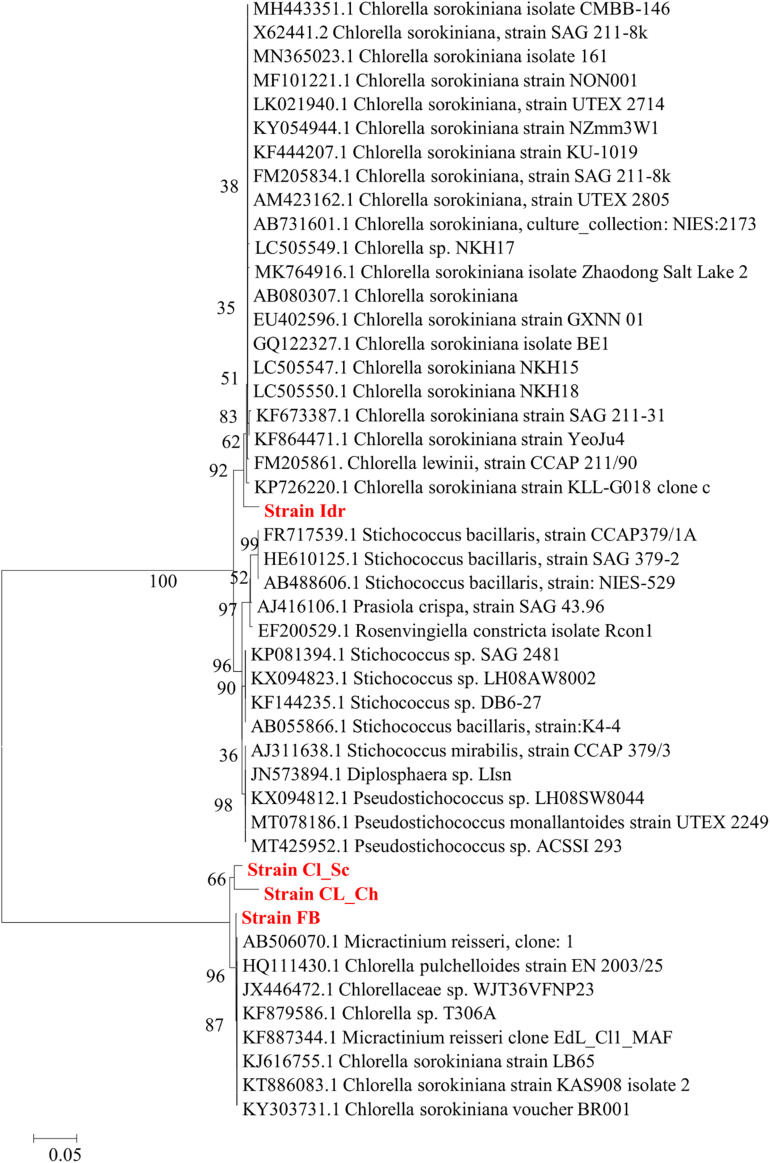
Phylogenetic tree reconstruction obtained with Neighbor Joining method on a total of 43 high quality sequences selected from the most similar to the sequences obtained for the four strains analyzed in this work (Acc. N. MT992788-MT992791). Reconstruction was performed using Tamura-Nei Model, partial-deletion, Nearest-Neighbor Interchange Heuristic Method, and 500 bootstrap resampling to evaluate the robustness of the inferred trees.

Phylogenetic analysis performed on the base of blast results is shown in Figures 3, 4, respectively for Maximum Likelihood and Neighbor Joining algorithms. In both cases strain sample Idr is separated from the other three strains in the phylogenetic trees. In both topologies, three different clades are distinguishable: one comprising most of the *C. sorokiniana* strains, a second clade comprising most *Stichococcus* sp. strain, and a third clade hosting different strains belonging to *C. sorokiniana*, *Chlorella* sp., *Micractinium reisserii*, and *C. pulchelloides* species. In both Maximum Likelihood and Neighbor Joining topologies, strain Idr is strictly associated with the *C. sorokiniana* clade and shows a basal position in Neighbor Joining. The other three strains are all together grouped in the last “multispecies” clade, that is anyway “dominated” by *C. sorokiniana* species; in the Maximum Likelihood topology strain “FB is strictly associated with a *Chlorella sorokiniana* (Acc. Nr.KT886083.1), while in Neighbor Joining topology it is associated with a 87% bootstrap support with all other strains forming the clade and belonging to different species. In both trees, strains CL_Sc and CL_Ch are grouped together within the “multispecies” clade.

### Physiological Parameters

In all the performed tolerance tests, the strains were able to grow in 1 week and in the chosen laboratory conditions in absence of every drug/test. The parameters compared to assess the growth in 1 week were O.D. (750 nm), total chlorophyll content and carotenoids. To this purpose, comparison between algal cultures at the beginning of the experiment (T0) and after 1 week (C), highlights the growth of all the strains in each test (Supplementary Figure 1).

### Metal Tolerance

**FIGURE 5:**
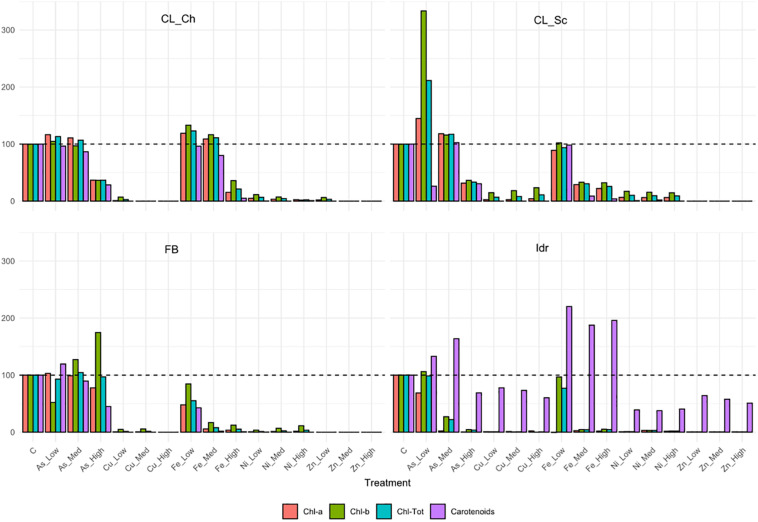
Pigment contents related to the metal tolerance tests, expressed as % compared to the pigment content of control test (C) in the algal strains. The 100% pigment content of C is represented by the dashed line.

The four microalgal strains show different tolerance patterns in presence of the same metal concentrations (Figure 5). The less tolerant strain is Idr, which is able to grow only in presence of As 0.5 mM, while the most tolerant in presence of different metals at different concentrations is FB, growing on both As (all concentrations) and Fe (2.5 mM). All strains are able to grow in presence of arsenite 0.5 mM; strain FB is able to grow up to the highest As concentration with a similar response than in control; CL_Ch and CL_Sc show similar patterns in presence of As, growing very well up to 1 mM, and reducing their growth of about a half compared to the control in presence of As 2.5 mM. CL_Ch is the only strain among the four growing up to 5 mM iron with similar photosynthetic pigment content than the control test; CL_Sc can grow on Fe-Med and Fe-High with a reduced rate. Interestingly, all the tests performed on strain Idr show high levels of carotenoids, a characteristic that is not shared by the other strains. Total chlorophyll and carotenoids amounts (Supplementary Figure 2) reflect the trends of Figure 5; indeed, in CL_Ch strain no statistically significant decrease is observed in presence of As up to 1 mM and in presence of Fe up to 5 mM, both for chlorophyll and for carotenoids. For FB strain no differences in chlorophyll content are observed at all the tested As concentrations while in all other tests the pigment amount significantly decrease, thus indicating growth difficulties in presence of metals. In Idr strain, all the tests result in a significant decrease in chlorophyll content except for As-Low, that remains at comparable levels than C. Finally, in CL_Sc, as in Fe-Low, the chlorophyll amount is similar than in C, in As-Low test it increases significantly, while in all other tests is significantly lower. Similar results for this strain are observed considering carotenoids that significantly decrease in all the tests but for As-Low and Fe-Low. Significant raise in carotenoids content was measured in strain Idr in presence of all iron concentrations (Supplementary Figure 2). O.D. measures of Ni tests reveal in all the four microalgal strains a statistically significant reduction of O.D. values if compared to C. Concerning As, in all the four strains O.D. values in presence of 0.5 mM arsenite is comparable to that of C. Finally, in CL_Sc, also As-Med concentration shows similar values than the C (Supplementary Figure 3).

### Tolerance Toward Herbicides and Ibuprofen

**FIGURE 6:**
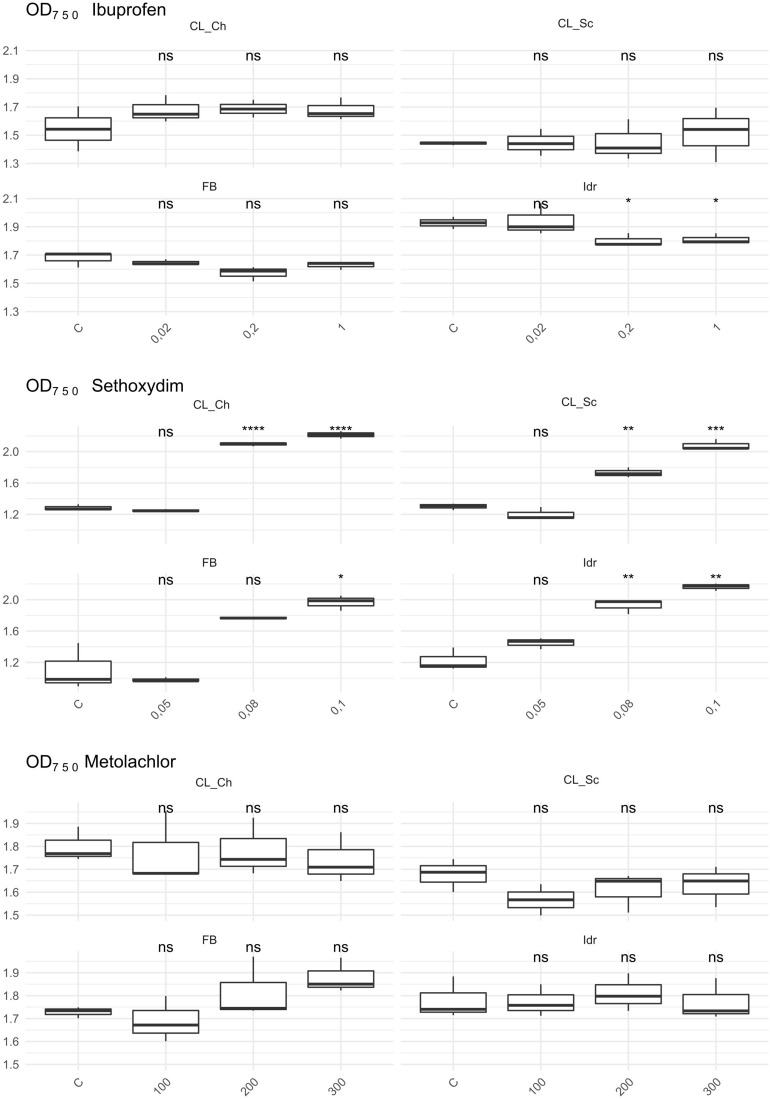
OD 750 value measured after 1 week exposure at herbicides and Ibuprofen. *T*-test has been applied to compare the effect of the presence of the substances with the control (C). The following convention for symbols indicating statistical significance were used: ns: *p* > 0.05; *: *p* ≤ 0.05; **: *p* ≤ 0.01; ***: *p* ≤ 0.001; ****: *p* ≤ 0.0001.

The first growth parameter that was analyzed, in order to check whether the four microalgal strains are able to grow in presence of herbicides (Metolachlor and Sethoxydim) and in presence of the common polluting drug Ibuprofen, was the O.D. (Figure 6). The presence of Metolachlor at all the tested concentrations seems to not affect the O.D. values of the microalgal strains in comparison to C. Idr strain is the only one negatively affected by the presence of Ibuprofen at 0.2 and 1 mg l^–1^ concentration. Finally, the intermediate and the high concentration of sethoxydim show higher O.D. values in all microalgal strains respect to C.

**FIGURE 7:**
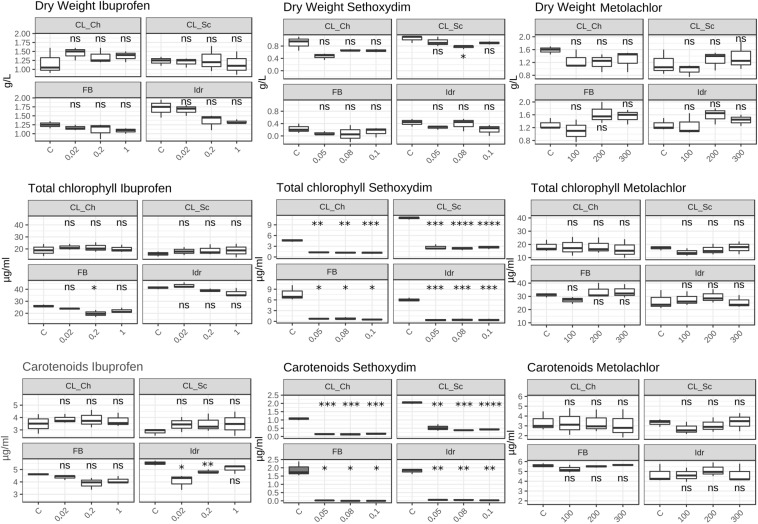
Photosynthetic pigment content and dry weight values measured in herbicides and Ibuprofen tests after 1 week exposure. Upper line: dry weight (mg l^–1^); middle line: total chlorophyll content (μg ml^–1^); lower line: total carotenoids content (μg ml^–1^). *T*-test has been applied to compare the effect of the presence of the substances with the control (C). The following convention for symbols indicating statistical significance were used: ns: *p* > 0.05; ^∗^: *p* ≤ 0.05; ^∗∗^: *p* ≤ 0.01; ^∗∗∗^: *p* ≤ 0.001; ^****^: *p* ≤ 0.0001.

The quantification of the photosynthetic pigment content (Figure 7) reveals different patterns of the four strains in presence of the different substances. While in presence of Metolachlor no statistically significant changes in pigment contents were measured respect to the C for all the strains, in presence of Sethoxydim at all the tested concentrations, significant decreases in both total chlorophyll and carotenoids content were observed. In presence of Ibuprofen, only a slight decrease in total chlorophyll content was measured in FB at 0.2 mg l^–1^, and a significant decrease in carotenoids content in Idr strain at 0.02 and 0.2 mg l^–1^. The DW measured did not reveal significant differences between the control and the treatments exposed to the three drugs. The only exception is represented by a slight decrease in DW in CL_Sc strain exposed to 200 μg l^–1^ Metolachlor (Figure 7).

### Antibiotic Resistance

**FIGURE 8:**
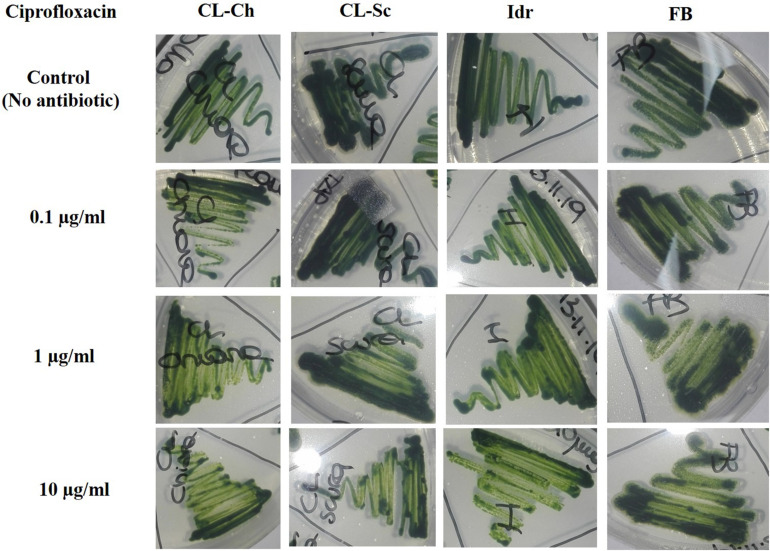
Microalgal growth in presence of different concentrations of Ciprofloxacin (antibiotic substance) in solid agar medium after 7 days growth.

**FIGURE 9:**
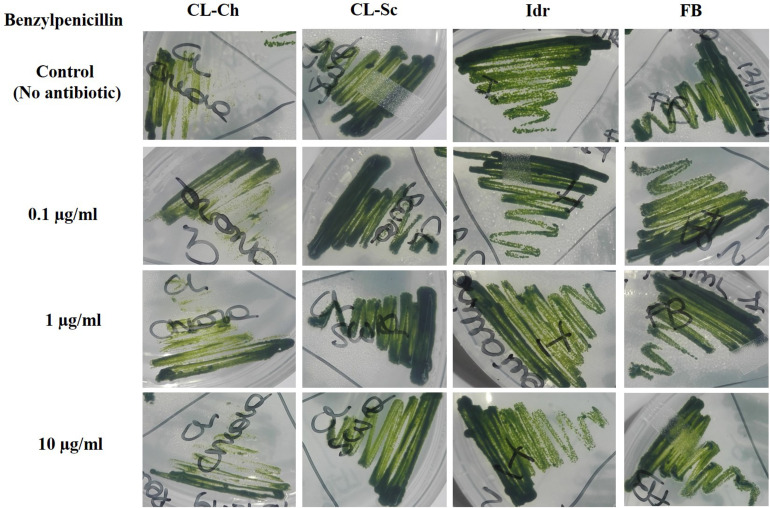
Microalgal growth in presence of different concentrations of Benzylpenicillin (antibiotic substance) in solid agar medium after 7 days growth.

All the four microalgal strains are able to grow in presence of the three Ciprofloxacin concentrations analogously to the control test in absence of the drug (Figure 8). The same can be assessed for Benzylpenicillin, with the only exception of strain CL_Ch that, at 10 μg l^–1^ Benzylpenicillin concentration, shows a slightly reduced growth respect to the control (Figure 9).

## Discussion

The importance of native microalgal strains is highlighted from many authors (Abdelaziz et al., 2014); indeed native strains show a better adaptability to environmental conditions of their isolation area, especially in comparison to other commercial species (i.e., Pandey et al., 2019). In this study, native microalgae from the Lake Massaciuccoli have been examined for the first time; their tolerance pattern against the most common environmental contaminants have been highlighted, underlying their potential in local wastewater treatments (Tuscany area) with promising growth rates and performances. The four newly isolated microalgal strains from the Lake Massaciuccoli show different physiological characteristics and different responses in presence of all the tested pollutants, despite the fact that all of them seem to be related to *Chlorella sorokiniana* species (Supplementary Tables 1A–D). From a morphological viewpoint, the average cell diameter is different in the four strain, indicating that CL_Ch is the biggest one, and Idr is the smallest (Figure 2). All the tolerance test have been correctly performed since all the strains in all the tests grew in absence of any substance in their culture medium (control) and in the experimental conditions, as confirmed by the increase in O.D. and photosynthetic pigment content (Supplementary Figure 1). Interestingly, phylogenetic analysis reveal that they are divided into two distinct groups, one more closely related to *C. sorokiniana*, to which strain Idr is associated, and another one comprehending the other three strains, more closely associated to a phylogenetic group harboring different species (Figures 3, 4), but anyway “dominated” by *C. sorokiniana*. It is worth of noting that even if the four strains have been collected from the same environment, and even if they are all phylogenetically related to *C*. *sorokiniana*, their physiological characteristics and their responses in presence of different environmental contaminants are different. This result reinforces the intraspecific difference that were found not only at morphological scale, but also at physiological and phylogenetic scale. According to this observation, we can cite several studies on microalgal biodiversity demonstrating that different strains belonging to the same species can show high diversity at strain level (i.e., Manandhar-Shrestha and Hildebrand, 2013; Barry et al., 2015); this is true also for *Chlorella sorokiniana* (Rosenberg et al., 2014). Intrastrain diversity within a microalgal species might lead to a differentiation in terms of metabolic features, as for example the production of lipids in a strain respect to another, belonging to the same species (Carrier et al., 2018). This characteristic might represent an advantage for the selection of strain with a biotechnological interest. Moreover, it is important to focus on the fact that different environmental niches, that can be recognizable in the different part of the Lake Massaciuccoli in which we collected water samples (Figure 1), may lead to the microalgal speciation (Lundholm and Moestrup, 2006), that can result also in strain diversity. The speciation process may or may not be accompanied by morphological changes in microalgae (Rynearson and Armbrust, 2004). To this purpose, and according to many authors (e.g., Brand, 1981; Rynearson and Armbrust, 2004), it is important to point out that phytoplankton can show intraspecific genetic variation. All the strains analyzed in this research showed tolerance toward arsenite, at least at the lower concentration tested. Accordingly, the microalgal population seems to be not affected by the presence of this metal in terms of produced biomass (Supplementary Figure 3). This aspect is important because if the biomass does not decrease respect to C, the cells continue to grow in presence of arsenite, suggesting that they could present a mechanism of exclusion of the metal, or a mechanism of active accumulation of the metal. Anyway, in both cases, the cells are not affected by the presence of the contaminant. This aspect will be investigated in the next research, that will be addressed at highlighting the mechanisms of inclusion/exclusions of the metals, in those algae showing active growth in presence of the contaminant. This data are similar to the result obtained with other freshwater microalgal strains tested in the same conditions (Chiellini et al., 2020), and suggest a potential use of these microalgae in arsenic removal from the environment. The same observation can be done also for iron, at least at the lower tested concentration. Strain Idr appears the most sensitive to the tested metals compared to the other three strains; this observation is enforced by the fact that this strain has the highest values of carotenoids content in all the metal tests (Figure 5), especially in presence of iron (Supplementary Figure 2), thus suggesting it is somehow stressed by the presence of metal contaminants. At the same time, strain CL_Ch is the most tolerant to all the tested metals. The differential response of Idr strain compared to the other three strains is in agreement with the fact that it is phylogenetically isolated in both tree topologies respect to the other isolated strains. Interestingly, strain Idr is also the one showing the lower average cell diameter, a distinctive morphological feature respect to the other strains (Figure 2). Moreover, according to the geographic position of the sampling sites, Idr strain is the only one collected in proximity of the “idrovora” site, close to the intensive agricultural land and further from the lake than the other three strains (Figure 1). This observation might suggest that the taxonomic separation between the two groups is also correlated to distinct tolerance patterns in presence of some environmental contaminants, and to the physical separation due to the sampling location. Probably, we cannot exclude the possibility that Idr strain might be adapted to an environment rich in nutrients, due to the presence of agricultural runoff, and simultaneously poor in contaminant derived from human activities. This characteristic might have caused a reduction in tolerance to the tested environmental pollutants such as antibiotics and Ibuprofen; indeed, these last don’t derive from agricultural runoff but from anthropogenic activity and, probably, they are more difficult to find in this sampling site respect to the center of the lake. To this purpose, it is important to observe that in the Lake Massaciuccoli, there are three main sources of civil discharges (Cini, 1999): (i) one from the small city of Vecchiano, which discharges directly into the Barra channel; (ii) a second one from the treatment plant of Migliarino, which discharges into the agricultural area of Vecchiano, (iii) and a third one from a food industry which discharges into the Separator channel. Moreover, in addition to the nutrient load deriving from the Barra channel, another important contribution is associated with the Pantaneto channel, which collects the wastewater from the Massarosa treatment plant (Cini, 1999).

In the last decades, the widespread (ab)use of antibiotics for the prevention and treatment of microbial infections, but also for the promotion of animal and plant growth, has led to the dispersion of antibiotics and degraded related substances in the environment (Kummerer, 2009). This is a particularly important matter since common wastewater treatment plants are not designed for the efficient removal of such substances (Gobel et al., 2007). An increasing accumulation of antibiotic substances in the environment is still occurring and causing the diffusion of multidrug resistant bacterial strains, which consequently can compromise public health (Zhang et al., 2009). Ciprofloxacin, one of the most diffused antibiotics in human and veterinary medicine against gram-negative and gram-positive bacterial infections, has been widely detected in surface water, groundwater, and wastewater (Lapworth et al., 2012). In green microalgae, Ciprofloxacin concentrations in the range of 20–40 mg l^–1^ (or μg ml^–1^) can inhibit *Chlorella vulgaris* growth by 50%; since these concentrations are of the same order of the ones tested in this experiment, we can assess that our strains are more resistant than a *C. vulgaris* that is instead sensitive to such antibiotic substance. This is quite understandable if we consider a species-specific differentiation in antibiotic tolerance, since our strains are much closely related to *C. sorokiniana* than *C. vulgaris*. Ciprofloxacin can also cause ≥30% inhibition in *Chlorella pyrenoidosa* at 100–150 mg l^–1^ (or μg ml^–1^); moreover, at concentrations comprised within 55–111 mg l^–1^ (or μg ml^–1^) it can inhibit the 50% growth of *Chlamydomonas mexicana* (Miazek and Brozek-Pluska, 2019). Anyway, in these last examples, concentrations are much higher than those tested in our experiment (and in any case, higher than the ones commonly detected in environmental freshwater), and the investigated species are not related to our strains. Benzylpenicillin (penicillin G) is an antibiotic widely used for the treatment of the common bacterial infections including pneumonia, strep throat, syphilis, necrotizing enterocolitis, diphtheria, gas gangrene, leptospirosis, cellulitis, and tetanus (Stuart et al., 2009). It contains a 6-[(2-phenylacetyl)amino] moiety in its structure (Miazek and Brozek-Pluska, 2019), and its inhibitory effect against *Microcystis aeruginosa* growth has been demonstrated at 0.006 mg l^–1^ (Halling-Sorensen, 2000). This result is not in agreement with our observations, demonstrating that our strains, even if belonging to different genera/species, can tolerate much higher concentrations of this antibiotic. Additionally, Benzylpenicillin at 7114 mg l^–1^ was observed causing 50% toxicity to *Pseudokirchneriella subcapitata* growth (Havelkova et al., 2016), but this value is not comparable to our results since it is almost a hundred times higher than the concentrations that we applied. We can assess that all our strains were able to grow in presence of all the tested concentrations of both antibiotics; only strain CL_Ch showed a slightly reduce growth in presence of Benzylpenicillin. According to Xiong et al. (2017), the growth of *Chlamydomonas mexicana* was slightly influenced at Ciprofloxacin concentrations below 10 mg l^–1^, similarly to our CL_Ch strain, and significantly inhibited at higher antibiotics concentrations, during the whole experimental duration (11 days). In the study of Zuccato et al. (2005), the quantification of common pharmaceutical products in the effluents of nine urban sewage treatment plants highlighted an average ciprofloxacin amount of about 300 ng l^–1^, corresponding to 0.0003 mg l^–1^, that is a concentration significantly lower than the one tested in this screening. In a more recent research (Zuccato et al., 2010), the same authors quantified the presence of antibiotics in four water treatment plants in the North of Italy, and in two Italian rivers (the river Po and the river Arno). Results highlighted that in the effluents of the Varese treatment plant the amount of Ciprofloxacin was about 150 ng l^–1^, while in the rivers, the amount ranged from 8.8 ± 8 ng l^–1^ in the river Po and 19 ± 16 ng l^–1^ in the river Arno. At the same time, the amount of amoxicillin (penicillin) in the river Arno accounted for 5.7 ± 2.9 ng l^–1^. Probably, the lower ciprofloxacin concentration found in aquatic environments might depend on the fact that this substance is rapidly degraded by photolysis since it is highly susceptible to light (Bai and Acharya, 2017). On the light of the tolerance that our strain showed toward antibiotic concentrations even higher compared to the concentrations commonly found in Italian freshwater environments (Figures 8, 9), we could hypothesize the presence of a possible mechanism of the four strains for antibiotic removal; indeed, we are planning to investigate whether such mechanism could be the exclusion or the accumulation of the drug, in order to verify if they represent good candidates to be used in the removal of antibiotics in wastewater treatment.

Ibuprofen is an anti-inflammatory drug commonly used for its analgesic and antipyretic properties, that can be obtained without medical prescription. It has been previously demonstrated that Ibuprofen is stable in wastewater for 30 days (Khalaf et al., 2013), hence, since common wastewater treatment plants are not planned for the removal of pharmaceuticals (for a review see Yang et al., 2017), there is the increasing need to find alternative solution for the degradation and removal of such substance from water. The application of the diatom *Phaeodactylum tricornutum* represents a putative solution since both its living and dead biomass are efficient in removing up to 99.9% at initial concentration of 2 mg l^–1^ (Santaeufemia et al., 2018). The biomass of this microalga is a good and eco-friendly alternative for applications that require the removal of Ibuprofen from aqueous solutions. On the other side, Ibuprofen seems to have toxic effects on the freshwater *Scenedesmus rubescens*, causing sufferance symptoms (morphological and ultrastructural alterations) and loss of growth at concentration of 1000 μg l^–1^ (Moro et al., 2014), corresponding to 1 mg l^–1^, that is the highest concentration that we tested in this study. *Scenedesmus rubescens* showed decrease in chlorophyll content and increase of carotenoids, indicating a stressful condition induced by drug. This trend is not in agreement with the observation made in this study since no significant stressful signals (i.e., significant carotenoids increment) was observed in the four algae (Figure 7). This is true for FB, CL_Sc, and CL_Ch strains; on the other side, strain Idr seem to be moderately affected by the presence of Ibuprofen respect to the other strains; accordingly, O.D. values show a significant decrease respect to the control test in terms of biomass (Figure 6), as well as the quantification of carotenoids content (Figure 7). The different tolerance pattern of strain Idr somehow reflects the phylogenetic divergence of the two groups of sequences (Figures 3, 4), as observed with metal exposure. According to literature, *C. sorokiniana* has been described as contributing to Ibuprofen removal from wastewater (de Wilt et al., 2016), opening new perspectives for evaluating the use of our strains in wastewater treatment plant for the removal of pharmaceuticals too.

As described in King et al. (2017), the two types of toxicity in freshwater microalgae for metolachlor are the chronic toxicity and the acute toxicity. All the growth parameters evaluated in this work for our strains do not highlight a toxic/deleterious effect of metolachlor on the four microalgae (Figure 7). This might suggest a possible tolerance pattern of the strains to this herbicide, possibly due to an exclusion or to an accumulation mechanism that does not affect the growth of the algal population. It has been previously demonstrated a not toxic effect of metolachlor in *Chlorella kessleri* after 2 days exposure at 200 μg l^–1^ (Spoljarich et al., 2011), a nor toxic effect on *Chlamydomonas reinhardtii* after 4 days exposure at 227.6 μg l^–1^ (Fairchild et al., 1998) and in *Chlamydomonas* strain CC125 after 2 days exposure at 595.4 μg l^–1^ (Fischer et al., 2012), and a not toxic effect of metolachlor 1 μg l^–1^ on *Chlorella pyrenoidosa* after 1 day exposure (Liu and Xiong, 2009). Moreover, it has also been observed an absence of toxicity on *Scenedesmus obliquus* after 4 days exposure at 31.2 μg l^–1^ (Bian et al., 2009). All these data refer to comparable metolachlor concentrations to the ones tested in this work (100, 200, and 300 μg l^–1^); even if there are not available data regarding the reaction of *C. sorokiniana* in presence of such substance, our data suggest that our strains are tolerant to this herbicide.

Considering the tests performed on sethoxydim, it is worth of note that the solution has a milky white appearance and this characteristic might have affected the O.D. values collected in the experiment (Figure 6) that, indeed, appear significantly higher with the increase of the concentration tested in all the four microalgal strains. According to this observation, the results of the quantification of photosynthetic pigments confirm the influence of the solution appearance in the O.D. values; indeed, in all the four strains at all the tested concentration, a significant decrease in both chlorophyll and carotenoids content was measured, thus confirming a scarce tolerance of this herbicide also at the minimal tested concentrations. According to Smythers et al. (2019), 0.08% v/v of Poast on *Chlorella vulgaris*, a non-target organism, causes several cell damages just after 15 min exposure. Poast is an herbicide formulation containing 18% sethoxydim (Sevilla-Morán et al., 2017) and the concentration tested in our experiment (0.05–0.1 pure sethoxydim) are higher than the one tested in Smythers et al. (2019). The deleterious effects on *C. vulgaris*, anyway, seem to be not related to the presence of sethoxydim in Poast composition, but to the other additional substances in the formulation. Accordingly, also Nagai (2019) observed an almost absence of toxicity of sethoxydim on seven microalgal strains.

## Conclusion

Four native microalgal strains related to the *Chlorella sorokiniana* lineage have been isolated from the Lake Massaciuccoli (Tuscany, Italy) for the first time. They were tested toward a panel of common environmental contaminants, in order to screen their first response in terms of death or survival, after 1-week exposure. Even if the strains were taxonomically related to the same species and were isolated from the same environment, they showed different tolerance patterns in presence of metals, herbicides, antibiotics, and Ibuprofen. Interestingly, phylogenetic analysis revealed two distinct groups of organisms, the first one comprehending only strain Idr, and the other one comprehending CL_Sc, CL_Ch, and FB strains. The morphological aspects of the organisms revealed differences in cell size as well, with Idr showing the smallest diameter values and CL_Sc the highest; this feature reflects the tolerance pattern of the microalgal strains as well as the taxonomic position. According to our data, intraspecific variation was found at taxonomic, morphological and physiological viewpoints. CL_Sc was the most tolerant in presence of metals, especially arsenite and iron, while strain Idr was the most sensitive. All strains were sensitive to sethoxydim and tolerant to metolachlor at all the tested concentrations. Strain Idr was the most sensitive in presence of Ibuprofen considering carotenoids content and O.D., while strain FB was the most sensitive to this substance considering chlorophyll content. Finally, strain CL_Ch was the most sensitive to the highest Benzylpenicillin concentration tested. Resistance pattern of strain Idr in presence of Ibuprofen and metals (i.e., the highest carotenoid amounts measured) somehow reflects the phylogenetic “isolation” from all the other three strains. Moreover, its tolerance pattern also reflects the physical separation of such strain among the four sampling sites. Overall, all the strains showed interesting responses in presence of very high concentrations of the tested substances and, for this reason, they are worthy of attention as putative candidates for water remediation in wastewater treatment plants. Interestingly, they could be also used for *in loco* water remediation within the Lake Massaciuccoli, as they represent autochthonous species. Further experimentations will be addressed to the exploration of their decontamination abilities in field scale treatment plants, in order to define which of them could be applied at large scale.

## Data Availability Statement

The sequences obtained in this study can be found in NCBI online repositories (https://www.ncbi.nlm.nih.gov/genbank/). Sequences are deposited under the following accession numbers: MT992788, MT992789, MT992790, and MT992791.

## Author Contributions

AC and CC conceived the idea and the experimental design. AC collected and maintained in culture the strain and performed the physiological analysis and interpretation. SS performed optical microscopy observation. CC performed molecular analysis, phylogenetic reconstructions, and data interpretation. CC, LG, and AC interpreted the results. All authors wrote parts of the manuscript, contributed toward revision and final approval of the manuscript, and contributed to the final writing, editing, and acceptance.

## Conflict of Interest

The authors declare that the research was conducted in the absence of any commercial or financial relationships that could be construed as a potential conflict of interest.

## Supplementary Material

Supplementary Figure 1 Comparison between the OD750 values, total chlorophyll and carotenoids content of the beginning (T0) and of the end T1 (1-week) of the experiment for each microalgal strain. Standard error bars are reported in the figure.

Supplementary Figure 2 Total chlorophyll and carotenoids contents related to the metal tolerance tests, expressed as μg ml^–1^ of algal culture. *T*-test has been applied to compare the effect of the metal respect to the control (C). The following convention for symbols indicating statistical significance were used: ns: *p* > 0.05; *: *p* ≤ 0.05; **: *p* ≤ 0.01; ***: *p* ≤ 0.001; ****: *p* ≤ 0.0001.

Supplementary Figure 3 OD 750 value measured after 1 week exposure at As and Ni. *T*-test has been applied to compare the effect of the presence of the metal with the control (C). The following convention for symbols indicating statistical significance were used: ns: *p* > 0.05; *: *p* ≤ 0.05; **: *p* ≤ 0.01; ***: *p* ≤ 0.001; ****: *p* ≤ 0.0001.

## References

[B1] Abdelaziz A. E. Leite G. B. Belhaj M. A. Hallenbeck P. C. (2014). Screening microalgae native to Quebec for wastewater treatment and biodiesel production. *Biores. Technol.* 157 140–148. 10.1016/j.biortech.2014.01.114 24549235

[B2] Ahmad A. L. Yasin N. M. Derek C. J. C. Lim J. K. (2011). Microalgae as a sustainable energy source for biodiesel production: a review. *Renew. Sust. Energ. Rev.* 15 584–593. 10.1016/j.rser.2010.09.018

[B3] Ali H. Khan E. Sajad M. A. (2013). Phytoremediation of heavy metals—concepts and applications. *Chemosphere* 91 869–881. 10.1016/j.chemosphere.2013.01.075 23466085

[B4] Altschul S. F. Madden T. L. Schaffer A. A. Zhang J. Zhang Z. Miller W. (1997). Gapped BLAST and PSI-BLAST: a new generation of protein database search programs. *Nucl. Acids Res.* 25 3389–3402. 10.1093/nar/25.17.3389 9254694 PMC146917

[B5] Amengual-Morro C. Niell G. M. Martínez-Taberner A. (2012). Phytoplankton as bioindicator for waste stabilization ponds. *J. Environ. Manage.* 95 S71–S76. 10.1016/j.jenvman.2011.07.008 21820796

[B6] Bai X. Acharya K. (2017). Algae-mediated removal of selected pharmaceutical and personal care products (PPCPs) from Lake Mead water. *Sci. Total Environ.* 581 734–740. 10.1016/j.scitotenv.2016.12.192 28089530

[B7] Barry A. N. Starkenburg S. R. Sayre R. T. (2015). Strategies for optimizing algal biology for enhanced biomass production. *Front. Energy Res.* 3:1 10.3389/fenrg.2015.00001

[B8] Bertacchi A. Giannini V. Di Franco C. Silvestri N. (2019). Using unmanned aerial vehicles for vegetation mapping and identification of botanical species in wetlands. *Landsc. Ecol. Eng.* 15 231–240. 10.1007/s11355-018-00368-1

[B9] Bian H. Chen J. Cai X. Liu P. Wang Y. Huang L. (2009). Dechlorination of chloroacetanilide herbicides by plant growth regulator sodium bisulfite. *Water Res.* 43 3566–3574. 10.1016/j.watres.2009.05.002 19520412

[B10] Boelee N. C. Temmink H. Janssen M. Buisman C. J. N. Wijffels R. H. (2011). Nitrogen and phosphorus removal from municipal wastewater effluent using microalgal biofilms. *Water Res.* 45 5925–5933. 10.1016/j.watres.2011.08.044 21940029

[B11] Boutin C. Lee H. B. Peart E. T. Batchelor P. S. Maguire R. J. (2000). Effects of the sulfonylurea herbicide metsulfuron methyl on growth and reproduction of five wetland and terrestrial plant species. *Environ. Toxicol. Chem.* 19 2532–2541. 10.1002/etc.5620191020

[B12] Brand L. E. (1981). Genetic variability in reproduction rates in marine phytoplankton populations. *Evolution* 35 1117–1127. 10.2307/2408125 28563400

[B13] Carrier G. Baroukh C. Rouxel C. Duboscq-Bidot L. Schreiber N. Bougaran G. (2018). Draft genomes and phenotypic characterization of Tisochrysis lutea strains. Toward the production of domesticated strains with high added value. *Algal Res.* 29 1–11. 10.1016/j.algal.2017.10.017

[B14] Chekroun K. B. Sánchez E. Baghour M. (2014). The role of algae in bioremediation of organic pollutants. *J. Iss. ISSN* 2360:8803.

[B15] Chiellini C. Guglielminetti L. Pistelli L. Ciurli A. (2020). Screening of trace metal elements for pollution tolerance of freshwater and marine microalgal strains: Overview and perspectives. *Algal Res.* 45:101751 10.1016/j.algal.2019.101751

[B16] Chiellini C. Iannelli R. Modeo L. Bianchi V. Petroni G. (2012). Biofouling of reverse osmosis membranes used in river water purification for drinking purposes: analysis of microbial populations. *Biofouling* 28 969–984. 10.1080/08927014.2012.724679 22971211

[B17] Chiellini C. Lombardo K. Mocali S. Miceli E. Fani R. (2019). *Pseudomonas* strains isolated from different environmental niches exhibit different antagonistic ability. *Ethol. Ecol. Evol.* 31 399–420. 10.1080/03949370.2019.1621391

[B18] Chiellini C. Miceli E. Bacci G. Fagorzi C. Coppini E. Fibbi D. (2018). Spatial structuring of bacterial communities in epilithic biofilms in the Acquarossa river (Italy). *FEMS Microbiol. Ecol.* 94:fiy181. 10.1093/femsec/fiy181 30202963

[B19] Cini C. (1999). *La distribuzione dei nutrienti nel bacino del lago di Massaciuccoli.* Lago di Massaciuccoli: verso il risanamento, 124–152.

[B20] Ciurli A. Zuccarini P. Alpi A. (2009). Growth and nutrient absorption of two submerged aquatic macrophytes in mesocosms, for reinsertion in a eutrophicated shallow lake. *Wetl. Ecol. Manag.* 17 107–115. 10.1007/s11273-008-9091-9

[B21] Cohen Z. (1999). *Chemicals from microalgae.* Florida: CRC Press.

[B22] Costa J. A. V. de Morais M. G. (2013). *16 Microalgae for Food Production.* Boca Raton: CRC Press, 405.

[B23] de Wilt A. Butkovskyi A. Tuantet K. Leal L. H. Fernandes T. V. Langenhoff A. (2016). Micropollutant removal in an algal treatment system fed with source separated wastewater streams. *J. Hazard. Mat.* 304 84–92. 10.1016/j.jhazmat.2015.10.033 26546707

[B24] Di Baccio D. Pietrini F. Bertolotto P. Pérez S. Barcelò D. Zacchini M. (2017). Response of Lemna gibba L. to high and environmentally relevant concentrations of ibuprofen: Removal, metabolism and morpho-physiological traits for biomonitoring of emerging contaminants. *Sci. Total Environ.* 584 363–373. 10.1016/j.scitotenv.2016.12.191 28104333

[B25] Duruibe J. O. Ogwuegbu M. O. C. Egwurugwu J. N. (2007). Heavy metal pollution and human biotoxic effects. *Int. J. Phys. Sci.* 2 112–118. 10.5897/IJPS.9000289

[B26] Fairchild J. F. Ruessler D. S. Carlson A. R. (1998). Comparative sensitivity of five species of macrophytes and six species of algae to atrazine, metribuzin, alachlor, and metolachlor. *Environ. Toxicol. Chem.* 17 1830–1834. 10.1002/etc.5620170924

[B27] Fischer B. B. Roffler S. Eggen R. I. L. (2012). Multiple stressor effects of predation by rotifers and herbicide pollution on different Chlamydomonas strains and potential impacts on population dynamics. *Environ. Toxicol. Chem.* 31 2832–2840. 10.1002/etc.2010 22996994

[B28] Gobel A. McArdell C. S. Joss A. Siegrist H. Giger W. (2007). Fate of sulfonamides, macrolides, and trimethoprim in different wastewater treatment technologies. *Sci. Total Environ.* 372 361–371. 10.1016/j.scitotenv.2006.07.039 17126383

[B29] Gonçalves A. L. Pires J. C. Simões M. (2017). A review on the use of microalgal consortia for wastewater treatment. *Algal Res.* 24 403–415. 10.1016/j.algal.2016.11.008

[B30] González-Barreiro O. Rioboo C. Herrero C. Cid A. (2006). Removal of triazine herbicides from freshwater systems using photosynthetic microorganisms. *Environ. Pollut.* 144 266–271. 10.1016/j.envpol.2005.12.014 16488522

[B31] Gorman D. S. Levine R. P. (1965). Cytochrome f and plastocyanin: their sequence in the photosynthetic electron transport chain of Chlamydomonas reinhardi. *PNAS* 54, 1665–1669. 10.1073/pnas.54.6.1665 4379719 PMC300531

[B32] Griffiths M. J. Garcin C. van Hille R. P. Harrison S. T. (2011). Interference by pigment in the estimation of microalgal biomass concentration by optical density. *J. Microbiol. Meth.* 85 119–123. 10.1016/j.mimet.2011.02.005 21329736

[B33] Hall T. A. (1999). BioEdit: a user-friendly biological sequence alignment editor and analysis program for Windows 95/98/NT. *Nucl. Acid. S.* 41 95–98.

[B34] Halling-Sorensen B. (2000). Algal toxicity of antibacterial agents used in intensive farming. *Chemosphere* 40 731–739. 10.1016/S0045-6535(99)00445-2 10705551

[B35] Havelkova B. Beklova M. Kovacova V. Hlavkova D. Pikula J. (2016). Ecotoxicity of selected antibiotics for organisms of aquatic and terrestrial ecosystems. *Neuro Endocrinol. Lett.* 37 38–44. 28263529

[B36] Khalaf S. Al-Rimawi F. Khamis M. Zimmerman D. Shuali U. Nir S. (2013). Efficiency of advanced wastewater treatment plant system and laboratory-scale micelle-clay filtration for the removal of ibuprofen residues. *J. Environ. Sci. Heal. B* 48 814–821. 10.1080/03601234.2013.781372 23688232

[B37] King O. Smith R. Mann R. Warne M. (2017). *Proposed aquatic ecosystem protection guideline values for pesticides commonly used in the Great Barrier Reef catchment area: Part 1–2, 4-D, Ametryn, Diuron, Glyphosate, Hexazinone, Imazapic, Imidacloprid, Isoxaflutole, Metolachlor, Metribuzin, Metsulfuron-methyl, Simazine and Tebuthiuron.* Brisbane: Department of Environment and Science.

[B38] Kumar K. S. Dahms H. U. Won E. J. Lee J. S. Shin K. H. (2015). Microalgae–a promising tool for heavy metal remediation. *Ecotoxicol. Environ. Safe.* 113 329–352. 10.1016/j.ecoenv.2014.12.019 25528489

[B39] Kummerer K. (2009). Antibiotics in the aquatic environment—a review—part I. *Chemosphere* 75 417–434. 10.1016/j.chemosphere.2008.11.086 19185900

[B40] Lapworth D. J. Baran N. Stuart M. E. Ward R. S. (2012). Emerging organic contaminants in groundwater: a review of sources, fate and occurrence. *Environ. Pollut.* 163 287–303. 10.1016/j.envpol.2011.12.034 22306910

[B41] Lastrucci L. Dell’Olmo L. Foggi B. Massi L. Nuccio C. Vicenti C. (2017). Contribution to the knowledge of the vegetation of the Lake Massaciuccoli (northern Tuscany. Italy). *Plant Sociol.* 54 67–87. 10.7338/pls2017541/03

[B42] Leong Y. K. Chang J. S. (2020). Bioremediation of heavy metals using microalgae: Recent advances and mechanisms. *Biores. Technol.* 303:122886. 10.1016/j.biortech.2020.122886 32046940

[B43] Lichtenthaler H. K. (1987). “Chlorophylls and carotenoids: Pigments of photosynthetic biomembranes,”. *Methods in Enzymology*, eds Abelson J. Simon M. Verdine G. Pyle A. (Cambridge: Academic Press), 350–382. 10.1016/0076-6879(87)48036-1

[B44] Liu H. Xiong M. (2009). Comparative toxicity of racemic metolachlor and S-metolachlor to Chlorella pyrenoidosa. *Aquat. Toxicol.* 93 100–106. 10.1016/j.aquatox.2009.04.006 19428127

[B45] Lundholm N. Moestrup Ø (2006). “The biogeography of harmful algae,” in *Ecology of harmful algae*, eds Turner J. T. Granéli E. (Berlin: Springer), 23–35. 10.1007/978-3-540-32210-8_3

[B46] Luo L. Wang P. Lin L. Luan T. Ke L. Tam N. F. Y. (2014). Removal and transformation of high molecular weight polycyclic aromatic hydrocarbons in water by live and dead microalgae. *Process Biochem.* 49 1723–1732. 10.1016/j.procbio.2014.06.026

[B47] Macchioni F. Chelucci L. Torracca B. Prati M. C. Magi M. (2015). Fishes and their parasites in the water district of Massaciuccoli (Tuscany, Central Italy). *Vet. Ital.* 51 199–203. 10.12834/VetIt.230.733.1 26455372

[B48] Manandhar-Shrestha K. Hildebrand M. (2013). Development of flow cytometric procedures for the efficient isolation of improved lipid accumulation mutants in a Chlorella sp. microalga. *J. Appl. Phycol.* 25 1643–1651. 10.1007/s10811-013-0021-8 24273383 PMC3825614

[B49] Matamoros V. Rodríguez Y. (2016). Batch vs continuous-feeding operational mode for the removal of pesticides from agricultural run-off by microalgae systems: A laboratory scale study. *J. Hazard. Mater.* 309 126–132. 10.1016/j.jhazmat.2016.01.080 26882523

[B50] Medlin L. Elwood H. J. Stickel S. Sogin M. L. (1988). The characterization of enzymatically amplified 16S-like rRNA-coding regions. *Gene* 71 491–499. 10.1016/0378-1119(88)90066-2 3224833

[B51] Mengoni A. Maida I. Chiellini C. Emiliani G. Mocali S. Fabiani A. (2014). Antibiotic resistance differentiates Echinacea purpurea endophytic bacterial communities with respect to plant organs. *Res. Microbiol.* 165 686–694. 10.1016/j.resmic.2014.09.008 25283726

[B52] Miazek K. Brozek-Pluska B. (2019). Effect of PHRs and PCPs on microalgal growth, metabolism and microalgae-based bioremediation processes: a review. *Int. J. Molec. Sci.* 20:2492. 10.3390/ijms20102492 31137560 PMC6567089

[B53] Mori G. Mattioli M. Madoni P. Ricci N. (1998). The ciliate communities of different habitats of Lake Massaciuccoli (Tuscany): Species composition and distribution. *Ital. J. Zool.* 65 191–202. 10.1080/11250009809386746

[B54] Moro I. Matozzo V. Piovan A. Moschin E. Dalla Vecchia F. (2014). Morpho-physiological effects of ibuprofen on *Scenedesmus rubescens*. *Environ. Toxicol. Pharmacol.* 38, 379–387. 10.1016/j.etap.2014.06.005 25128768

[B55] Muller-Feuga A. (2013). “Microalgae for aquaculture: the current global situation and future trends,”. *Handbook of Microalgal Cultures: Applied Phycology and Biotechnology*, 2nd Edn, ed. Richmond A. (West Sussex: Wiley Blackwell), 615–627.

[B56] Nagai T. (2019). Sensitivity differences among seven algal species to 12 herbicides with various modes of action. *J. Pestic. Sci.* 44 D19–D039. 10.1584/jpestics.D19-039 32874124 PMC7458096

[B57] Oliveira R. C. Palmieri M. C. Garcia O. J. (2011). “Biosorption of metals: state of the art, general features, and potential applications for environmental and technological processes,” in *Progress in Biomass and Bioenergy Production*, ed. Shaukat S. S. (London: IntechOpen).

[B58] Pacheco D. Rocha A. C. Pereira L. Verdelhos T. (2020). Microalgae Water Bioremediation: Trends and Hot Topics. *Appl. Sci.* 10:1886 10.3390/app10051886

[B59] Pandey A. Srivastava S. Kumar S. (2019). Isolation, screening and comprehensive characterization of candidate microalgae for biofuel feedstock production and dairy effluent treatment: a sustainable approach. *Biores. Technol.* 293:121998. 10.1016/j.biortech.2019.121998 31473377

[B60] Petroni G. Dini F. Verni F. Rosati G. (2002). A molecular approach to the tangled intrageneric relationships underlying phylogeny in Euplotes (*Ciliophora. Spirotrichea*). *Mol. Phylogenet. Evol.* 22 118–130. 10.1006/mpev.2001.1030 11796035

[B61] Rai P. K. (2008). Heavy metal pollution in aquatic ecosystems and its phytoremediation using wetland plants: an ecosustainable approach. *Int. J. Phytoremed.* 10 133–160. 10.1080/15226510801913918 18709926

[B62] Rosenberg J. N. Kobayashi N. Barnes A. Noel E. A. Betenbaugh M. J. Oyler G. A. (2014). Comparative analyses of three Chlorella species in response to light and sugar reveal distinctive lipid accumulation patterns in the microalga *C. sorokiniana*. *PLoS One.* 9:e92460. 10.1371/journal.pone.0092460 24699196 PMC3974682

[B63] Rynearson T. A. Armbrust E. V. (2004). Genetic differentiation among population of the planktonic marine diatom Ditylum brightwelli (Bacillariophyceae). *J. Phycol.* 40 34–43. 10.1046/j.1529-8817.2004.03089.x

[B64] Saba F. Papizadeh M. Khansha J. Sedghi M. Rasooli M. Amoozegar M. A. (2016). A rapid and reproducible genomic DNA extraction protocol for sequence-based identification of archaea, bacteria, cyanobacteria, diatoms, fungi, and green algae. *J. Med. Bacteriol.* 2016 22–28.

[B65] Sabater S. Guasch H. Ricart M. Romaní A. Vidal G. Klünder C. (2007). Monitoring the effect of chemicals on biological communities. The biofilm as an interface. *Anal. Bioanal. Chem.* 387 1425–1434. 10.1007/s00216-006-1051-8 17225111

[B66] Santaeufemia S. Torres E. Abalde J. (2018). Biosorption of ibuprofen from aqueous solution using living and dead biomass of the microalga Phaeodactylum tricornutum. *J. Appl. Phycol.* 30 471–482. 10.1007/s10811-017-1273-5

[B67] Schwartzbach S. D. Shigeoka S. (eds) (2017). *Euglena: biochemistry, cell and molecular biology*, Vol. 979 Berlin: Springer.

[B68] Sevilla-Morán B. Calvo L. López-Goti C. Alonso-Prados J. L. Sandín-España P. (2017). Photodegradation behaviour of sethoxydim and its comercial formulation Poast^®^ under environmentally-relevant conditions in aqueous media. Study of photoproducts and their toxicity. *Chemosphere* 168 501–507. 10.1016/j.chemosphere.2016.11.026 27865884

[B69] Sheath R. G. Wehr J. D. (2015). “Introduction to the freshwater algae,” in *Freshwater Algae of North America*, eds Wehr J. D. Sheath R. G. Kociolek J. P. (Cambridge: Academic Press), 1–11. 10.1002/9780470689554.ch1

[B70] Smythers A. L. Garmany A. Perry N. L. Higginbotham E. L. Adkins P. E. Kolling D. R. (2019). Characterizing the effect of Poast on Chlorella vulgaris, a non-target organism. *Chemosphere* 219 704–712. 10.1016/j.chemosphere.2018.12.050 30557727

[B71] Spandre R. Meriggi A. (1997). Studio della circolazione idrica all’interno del padule e del lago di Massaciuccoli. *Mem. Soc. Tosc. Sc. Nat. Seria A* 103 1–15.

[B72] Spoljarich D. Cipak A. Horvatic J. Andrisic L. Waeg G. Zarkovic N. (2011). Endogenous 4-hydroxy-2-nonenal in microalga Chlorella kessleri acts as a bioactive indicator of pollution with common herbicides and growth regulating factor of hormesis. *Aquat. Toxicol.* 105 552–558. 10.1016/j.aquatox.2011.08.007 21937009

[B73] Stuart M. C. Kouimtzi M. Hill S. R. (eds) (2009). *WHO Model Formulary 2008.* Geneva: World Health Organization.

[B74] Tam N. F. Wong Y. S. (eds) (1997). *Wastewater treatment with algae.* Berlin: Springer-Verlag.

[B75] Tamura K. Peterson D. Peterson N. Stecher G. Nei M. Kumar S. (2011). MEGA5: molecular evolutionary genetics analysis using maximum likelihood, evolutionary distance, and maximum parsimony methods. *Mol. Biol. Evolut.* 28 2731–2739. 10.1093/molbev/msr121 21546353 PMC3203626

[B76] Ternes T. A. Joss A. Siegrist H. (2004). Scrutinizing pharmaceuticals and personal care products in wastewater treatment. *Environ. Sci. Technol.* 38 392A–399A. 10.1021/es040639t 15543724

[B77] Wang Y. Ho S. H. Cheng C. L. Guo W. Q. Nagarajan D. Ren N. Q. (2016). Perspectives on the feasibility of using microalgae for industrial wastewater treatment. *Biores. Technol.* 222 485–497. 10.1016/j.biortech.2016.09.106 27765375

[B78] Wickham H. (2009). *ggplot2: Elegant Graphics for Data Analysis.* New York: Springer-Verlag, 2009.

[B79] Xiong J. Q. Kurade M. B. Jeon B. H. (2018). Can microalgae remove pharmaceutical contaminants from water? *Trends Biotechnol.* 36 30–44. 10.1016/j.tibtech.2017.09.003 28993012

[B80] Xiong J. Q. Kurade M. B. Kim J. R. Roh H. S. Jeon B. H. (2017). Ciprofloxacin toxicity and its co-metabolic removal by a freshwater microalga Chlamydomonas mexicana. *J. Hazard. Mater.* 323 212–219. 10.1016/j.jhazmat.2016.04.073 27180206

[B81] Yadav K. K. Gupta N. Kumar V. Singh J. K. (2017). Bioremediation of heavy metals from contaminated sites using potential species: a review. *Indian J. Environ. Prot.* 37:65.

[B82] Yang Y. Ok Y. S. Kim K. H. Kwon E. E. Tsang Y. F. (2017). Occurrences and removal of pharmaceuticals and personal care products (PPCPs) in drinking water and water/sewage treatment plants: A review. *Sci.Total Environ.* 596 303–320. 10.1016/j.scitotenv.2017.04.102 28437649

[B83] Zhang X. X. Zhang T. Fang H. H. (2009). Antibiotic resistance genes in water environment. *Appl. Microbiol. Biotechnol.* 82 397–414. 10.1007/s00253-008-1829-z 19130050

[B84] Zuccato E. Castiglioni S. Fanelli R. (2005). Identification of the pharmaceuticals for human use contaminating the Italian aquatic environment. *J. Hazard. Mater.* 122 205–209. 10.1016/j.jhazmat.2005.03.001 15967275

[B85] Zuccato E. Castiglioni S. Bagnati R. Melis M. Fanelli R. (2010). Source, occurrence and fate of antibiotics in the Italian aquatic environment. *J. Hazard. Mater.* 179 1042–1048. 10.1016/j.jhazmat.2010.03.110 20456861

