# Supplementary figures and images for: Isolation of Four Microalgal Strains From the Lake Massaciuccoli: Screening of Common Pollutants Tolerance Pattern and Perspectives for Their Use in Biotechnological Applications
Source: Front Plant Sci. 2020 Dec 9;11:607651. doi: 10.3389/fpls.2020.607651 (PMC7756032; doi:10.3389/fpls.2020.607651)

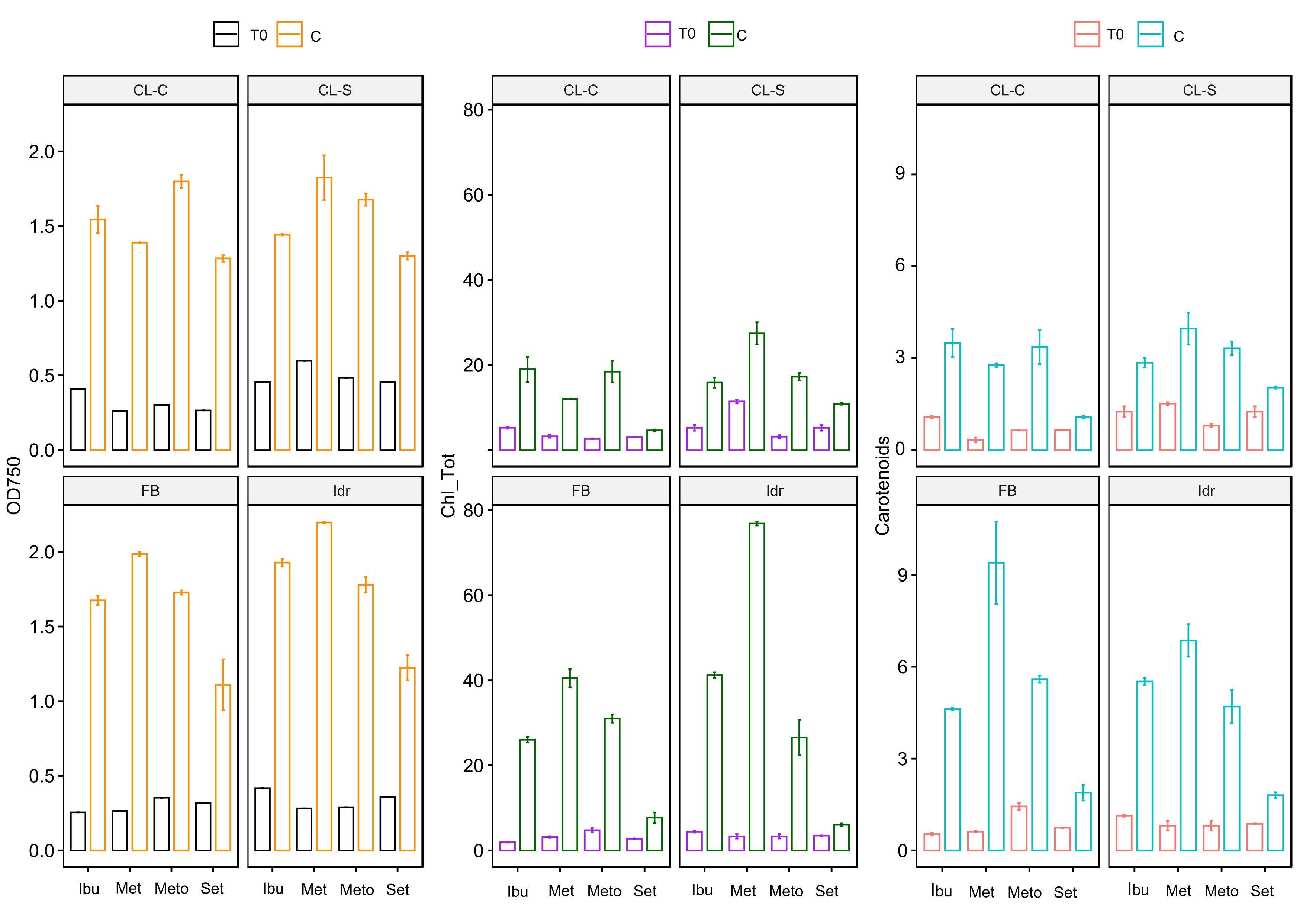

Supplement: Supplementary Figure 1 — Comparison between the OD750 values, total chlorophyll and carotenoids content of the beginning (T0) and of the end T1 (1-week) of the experiment for each microalgal strain. Standard error bars are reported in the figure. [file Image_1.JPEG]

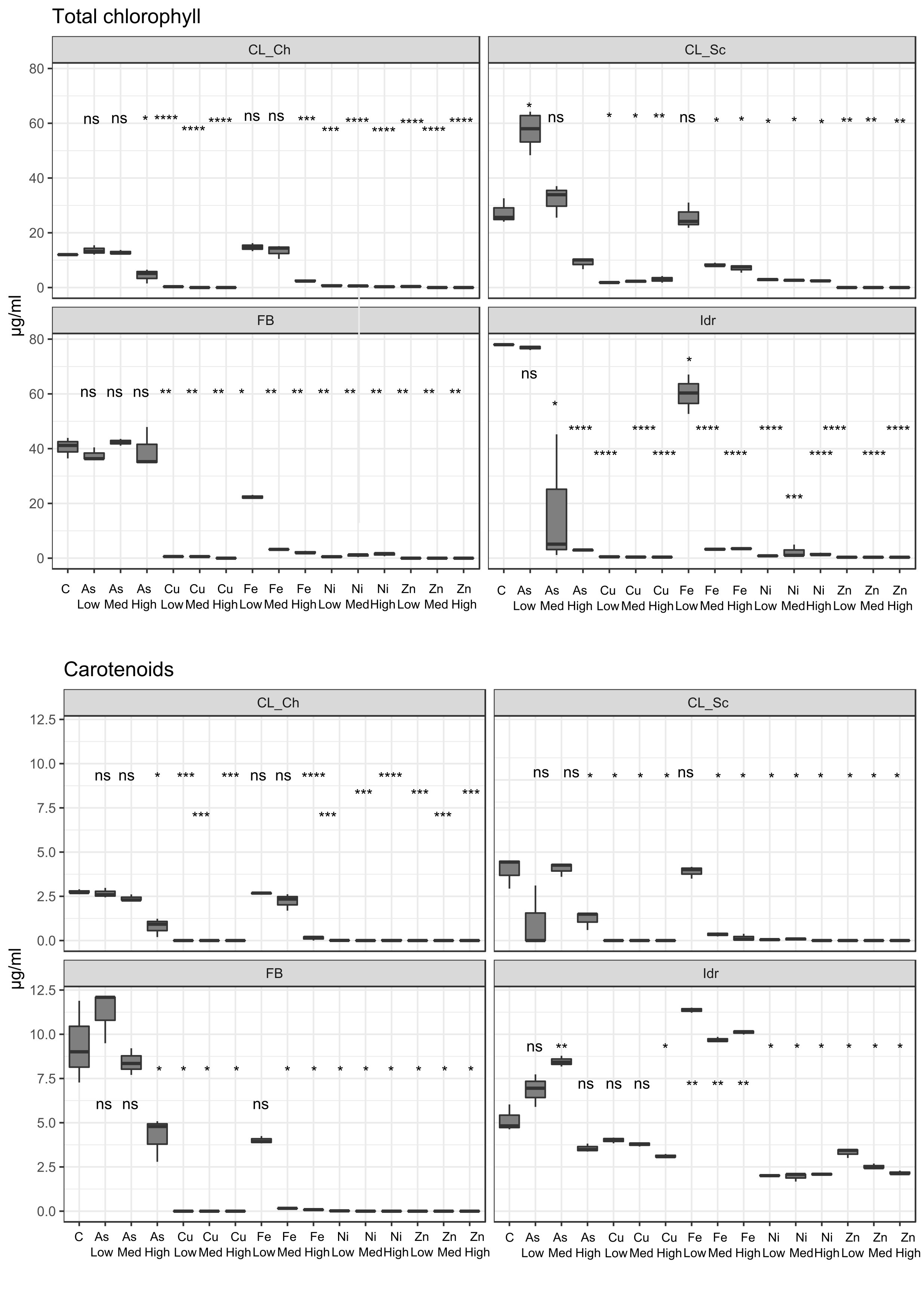

Supplement: Supplementary Figure 2 — Total chlorophyll and carotenoids contents related to the metal tolerance tests, expressed as μg ml–1 of algal culture. T-test has been applied to compare the effect of the metal respect to the control (C). The following convention for symbols indicating statistical significance were used: ns: p > 0.05; *: p ≤ 0.05; **: p ≤ 0.01; ***: p ≤ 0.001; ****: p ≤ 0.0001. [file Image_2.JPEG]

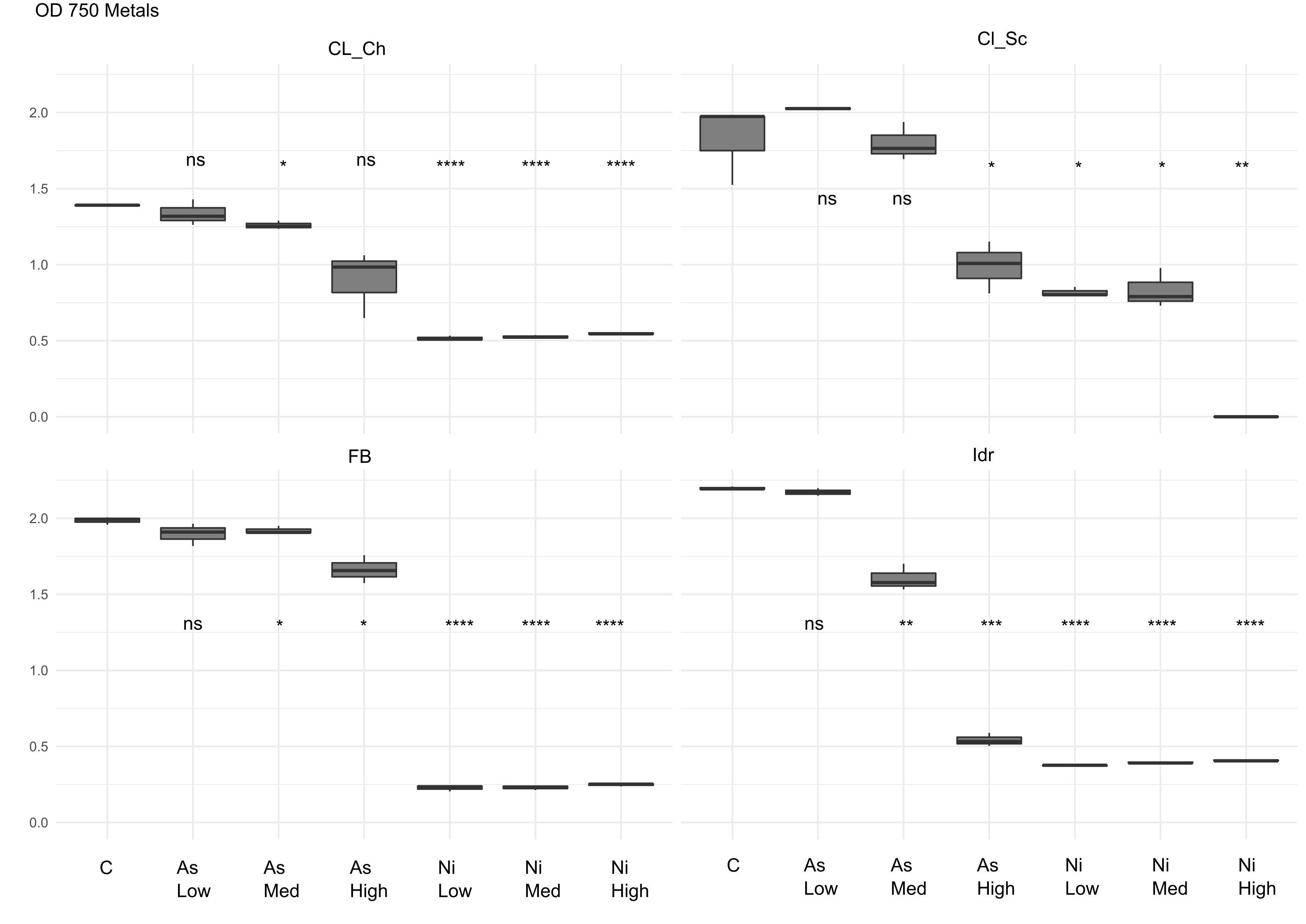

Supplement: Supplementary Figure 3 — OD 750 value measured after 1 week exposure at As and Ni. T-test has been applied to compare the effect of the presence of the metal with the control (C). The following convention for symbols indicating statistical significance were used: ns: p > 0.05; *: p ≤ 0.05; **: p ≤ 0.01; ***: p ≤ 0.001; ****: p ≤ 0.0001. [file Image_3.JPEG]
