# Supplementary material for: Isolation of Four Microalgal Strains From the Lake Massaciuccoli: Screening of Common Pollutants Tolerance Pattern and Perspectives for Their Use in Biotechnological Applications
Source: Front Plant Sci. 2020 Dec 9;11:607651. doi: 10.3389/fpls.2020.607651 (PMC7756032; doi:10.3389/fpls.2020.607651)
Supplement: Supplementary file 4 [file Table_1.DOCX]

Supplementary Tables 1a (CL_C), 1b (CL_Sc), 1c (FB) and 1d (Idr): NCBI blast analysis results of sequence 18S rRNA, showing the 10 closely related hits in public databases. Max Score: the highest alignment score calculated from the sum of the rewards for matched nucleotides and penalities for mismatches and gaps; Tot Score: the sum of alignment scores of all segments from the same subject sequence. Query Cover: the percent of the query length that is included in the aligned segments. Ident: the highest percent identity for a set of aligned segments to the same subject sequence.

| **1a: Strain CL-Ch similarities in NCBI Blast analysis** | | | | | |
| --- | --- | --- | --- | --- | --- |
| **Description** | **Accession** | **Max Score** | **Total Score** | **Query Cover** | **Per. Ident** |
| Chlorella sorokiniana strain MF1 | MN850520.1 | 1049 | 1049 | 95% | 95.03% |
| Chlorella sorokiniana NKH18 | LC505550.1 | 1049 | 1049 | 95% | 95.03% |
| Chlorella sp. NKH17 | LC505549.1 | 1049 | 1049 | 95% | 95.03% |
| Chlorella sorokiniana NKH15 | LC505547.1 | 1049 | 1049 | 95% | 95.03% |
| Micractinium sp. ACSSI 198 | MK235183.1 | 1049 | 1049 | 95% | 95.03% |
| Chlorella sorokiniana isolate 19-4 | KU948990.1 | 1049 | 1049 | 95% | 95.03% |
| Chlorella sorokiniana strain KAS908 isolate 2 | KT886083.1 | 1049 | 1049 | 95% | 95.03% |
| Chlorella sorokiniana strain KLL-G018 | KP726220.1 | 1049 | 1049 | 95% | 95.03% |
| Chlorella sorokiniana | KP771817.1 | 1049 | 1049 | 95% | 95.03% |
| Micractinium reisseri clone EdL_Cl1_MAF | KF887344.1 | 1049 | 1049 | 95% | 95.03% |

| **1b: Strain CL-Sc similarities in NCBI Blast analysis** | | | | | |
| --- | --- | --- | --- | --- | --- |
| **Description** | **Accession** | **Max Score** | **Total Score** | **Query Cover** | **Per. Ident** |
| Chlorella sorokiniana voucher BR001 | KY303731.1 | 1382 | 1382 | 99% | 98.02% |
| Chlorella sorokiniana NKH18 | LC505550.1 | 1381 | 1381 | 99% | 98.14% |
| Chlorella sp. NKH17 | LC505549.1 | 1381 | 1381 | 99% | 98.14% |
| Chlorella sorokiniana strain FZU60 | MK968764.1 | 1381 | 1381 | 99% | 98.14% |
| Chlorella sorokiniana isolate Zhaodong Salt Lake 2 | MK764916.1 | 1381 | 1381 | 99% | 98.14% |
| Micractinium sp. ACSSI 198 | MK235183.1 | 1381 | 1381 | 99% | 98.14% |
| Chlorella sorokiniana strain NZmm3W1 | KY054944.1 | 1381 | 1381 | 99% | 98.14% |
| Chlorella sorokiniana isolate 19-4 | KU948990.1 | 1381 | 1381 | 99% | 98.14% |
| Chlorella sorokiniana strain KAS908 isolate 2 | KT886083.1 | 1381 | 1381 | 99% | 98.14% |
| Chlorella sorokiniana strain KLL-G018 clone c | KP726220.1 | 1381 | 1381 | 99% | 98.14% |

| **1c: Strain FB similarities in NCBI Blast analysis** | | | | | |
| --- | --- | --- | --- | --- | --- |
| **Description** | **Accession** | **Max Score** | **Total Score** | **Query Cover** | **Per. Ident** |
| Chlorella sorokiniana NKH18 | LC505550.1 | 1434 | 1434 | 100% | 98.45% |
| Chlorella sp. NKH17 | LC505549.1 | 1434 | 1434 | 100% | 98.45% |
| Micractinium sp. ACSSI 198 | MK235183.1 | 1434 | 1434 | 100% | 98.45% |
| Chlorella sorokiniana isolate 19-4 | KU948990.1 | 1434 | 1434 | 100% | 98.45% |
| Chlorella sorokiniana strain KAS908 isolate 2 | KT886083.1 | 1434 | 1434 | 100% | 98.45% |
| Chlorella sorokiniana strain KLL-G018 clone c | KP726220.1 | 1434 | 1434 | 100% | 98.45% |
| Chlorella sorokiniana | KP771817.1 | 1434 | 1434 | 100% | 98.45% |
| Micractinium reisseri clone EdL_Cl1_MAF | KF887344.1 | 1434 | 1434 | 100% | 98.45% |
| Chlorella sp. GC | KF773743.1 | 1434 | 1434 | 100% | 98.45% |
| Chlorella sorokiniana | KJ149805.1 | 1434 | 1434 | 100% | 98.45% |

| **1d: Strain Idr similarities in NCBI Blast analysis** | | | | | |
| --- | --- | --- | --- | --- | --- |
| **Description** | **Accession** | **Max Score** | **Total Score** | **Query Cover** | **Per. Ident** |
| Chlorella sorokiniana NKH18 | LC505550.1 | 2840 | 2840 | 100% | 97.58% |
| Chlorella sorokiniana isolate 19-4 | KU948990.1 | 2840 | 2840 | 100% | 97.58% |
| Chlorella sp. GC | KF773743.1 | 2840 | 2840 | 100% | 97.58% |
| Chlorella sorokiniana, culture_collection: NIES:2173 | AB731602.1 | 2840 | 2840 | 100% | 97.58% |
| Chlorella sorokiniana, culture_collection: NIES:2173 | AB731601.1 | 2840 | 2840 | 100% | 97.58% |
| Chlorella sorokiniana isolate BE1 | GQ122327.1 | 2840 | 2840 | 100% | 97.58% |
| Chlorella sorokiniana, strain SAG 211-8k | FM205834.1 | 2840 | 2840 | 100% | 97.58% |
| Chlorella sorokiniana | AB080307.1 | 2840 | 2840 | 100% | 97.58% |
| Chlorella sorokiniana, strain SAG 211-8k | X62441.2 | 2837 | 2837 | 100% | 97.52% |
| Micractinium sp. ACSSI | MK235183.1 | 2836 | 2836 | 100% | 97.52% |
